# A beginner’s guide to manual curation of transposable elements

**DOI:** 10.1186/s13100-021-00259-7

**Published:** 2022-03-30

**Authors:** Clement Goubert, Rory J. Craig, Agustin F. Bilat, Valentina Peona, Aaron A. Vogan, Anna V. Protasio

**Affiliations:** 1 Canadian Center for Computational Genomics, McGill University, Montreal, Québec Canada; 2 Department of Human Genetics, McGill University, Montreal, Québec Canada; 3 Institute of Evolutionary Biology, University of Edinburgh, Edinburgh, EH9 3FL UK; 4 Departamento de Genética, Facultad de Medicina, Universidad de la República, Montevideo, Uruguay; 5 Department of Organismal Biology, Uppsala University, Norbyvägen 18D, 752 36 Uppsala, Sweden; 6 Department of Pathology, Tennis Court Road, Cambridge, CB1 2PQ UK; 7 Christ’s College, St Andrews Street, Cambridge, CB2 3BU UK

## Abstract

**Background:**

In the study of transposable elements (TEs), the generation of a high confidence set of consensus sequences that represent the diversity of TEs found in a given genome is a key step in the path to investigate these fascinating genomic elements. Many algorithms and pipelines are available to automatically identify putative TE families present in a genome. Despite the availability of these valuable resources, producing a library of high-quality full-length TE consensus sequences largely remains a process of manual curation. This know-how is often passed on from mentor-to-mentee within research groups, making it difficult for those outside the field to access this highly specialised skill.

**Results:**

Our manuscript attempts to fill this gap by providing a set of detailed computer protocols, software recommendations and video tutorials for those aiming to manually curate TEs. Detailed step-by-step protocols, aimed at the complete beginner, are presented in the Supplementary Methods.

**Conclusions:**

The proposed set of programs and tools presented here will make the process of manual curation achievable and amenable to all researchers and in special to those new to the field of TEs.

**Supplementary Information:**

The online version contains supplementary material available at 10.1186/s13100-021-00259-7.

## Introduction

Transposable elements (TEs) are mobile genetic entities generally found in multiple copies in the genome. They are ubiquitous across life, highly diverse, and can occupy large proportions of many eukaryotic genomes; for example, ~ 50% of the human genome is derived from TEs [1]. Despite their ubiquity, TEs have historically been understudied in genomic analyses, partly stemming from their incomplete representation in assemblies produced from short-read sequencing. However, with the wide-ranging importance of TE biology attracting greater recognition and many more genomes now being assembled to high-standards following the advent of long-read sequencing technologies, researchers are increasingly paying more attention to the repetitive fraction of genomes.

TE identification has become an intrinsic part of genome projects and, in line with this, many de novo and homology-based algorithms have been developed (refs [2–6]. to name a few). Although these algorithms have dramatically improved our capacity to identify TEs and other genomic repetitive sequences, in most cases they lack the exactitude required for certain downstream applications. For example, while automated repeat identification may be sufficient for general repeat masking (e.g. prior to gene annotation), detailed analysis of TE diversity and evolution within a genome generally requires greater accuracy [7]. In particular, with the aim of trying to identify as many TE candidate sequences as possible, automated tools are often greedy and report a number of chimeras (i.e. a fusion of two distinct TEs) that may appear only once or twice in the genome. This is not a criticism of the tools themselves! The complexity of TE biology and eukaryotic genomes makes developing the perfect TE prediction tool, where no family is missing and all start and end sites are well defined, incredibly challenging. Therefore, until such a perfect tool exists researchers need to dedicate time and resources to manually curate or inspect the output of automated prediction tools [8, 9]. In summary, it is accepted among the TE scientific community that a substantial amount of manual curation is required to arrive at a highly reliable set of TE consensus sequences, normally called a “transposable element consensus library” or “TE library” (see Glossary).

The process of manual curation is laborious and time consuming, but so far unavoidable if producing a “gold standard” TE library is desirable. Platt II et al. [7] showed that relying solely on automated methods of TE detection is insufficient to fully characterise the TE content of an organism’s genome. The authors demonstrated that it is possible to use homology-based methods, i.e. annotation using a TE reference library from a closely related species, to achieve a good approximation of the fraction of the genome covered by TEs. However, they also found that this approach often results in inaccurate calculations of TE divergence, making many TE families from the newly annotated genome appear to have high levels of divergence and hence be inferred as much “older”. This happens simply because the divergence parameter is calculated in relation to the reference TE which in this case comes from a related, yet not the same, species. Manual curation may also be needed if the end point of the analyses is to characterise the full content and diversity of TEs with a focus on the different superfamilies, distance relationships among families of the same superfamily, etc. A well curated library based on the actual genome of interest may lead to very different conclusions, especially for TE families that have been recently active. Furthermore, homology-based approaches are only applicable if TE libraries from related species are available, which increasingly is not the case as more diverse branches of the eukaryotic tree are targeted for sequencing.

Platt II et al. [7] published, alongside their manuscript, a set of scripts and recommendations to aid the manual curation process [7] and these have been adopted by many others (refs [8–10] to name a few). More recently, Storer et al. [11] published an excellent set of protocols and scripts to aid manual curation. Because of the labour intensive nature of this process and the lack of a general protocol for manual curation, this knowledge is often passed on orally from mentor to mentee, making it difficult for those “outside” the field to initiate the process of manually curating the TE content of their favourite genome. However, a step-by-step guide that describes algorithms alongside structural characteristics of TEs and the “know how” of the manual curation process is so far missing from the literature.

In this work, we aim to bridge the gap between automated annotation of TEs and the generation of a manually curated library by providing a set of guidelines and suggestions to carry out this process. The protocols are intended to be used by beginners as well as trained bioinformaticians. We assume the reader has some basic knowledge of the command line computing system (that is Unix/Linux), although suggestions for gaining this knowledge are also provided in the Supplementary Methods. All of the steps described here can be carried out on a local computer or laptop, although access to a computer cluster or high-performance computer can be beneficial.

## Methods

The most likely start of the manual curation process is after having run one or several de novo TE prediction algorithms on the interrogated genome assembly. A review of these methods is outside the scope of this work, but a semi-comprehensive list can be found in the community resource TEhub.org [12]. Depending on the size of the genome to be analysed, de novo TE prediction algorithms may be run on a computer cluster or server rather than on a desktop or laptop computer (see Supplementary Methods for an alternative to computer servers). One of the most widely used pipelines is RepeatModeler2 [6]. This pipeline uses a number of different algorithms including LTR_harvest [13], RepeatScout [5] and Recon [4] to predict putative TE families, providing as an output a set of consensus sequences in FASTA format. Alternatively, the starting point of the manual curation process could be a highly repetitive and possibly unknown sequence in a given genome or a smaller set of TE families of interest rather than a comprehensive set of putative TE families. In any case, this workflow should be able to assist the manual curation process.

In the next sections, the reader is presented with a series of bioinformatics tools that will take them from a prospective TE sequence, through the search of similar sequences in a genome or database, the generation of a multiple sequence alignment, the manual curation of said alignment, and finally generation of a TE consensus. The ultimate goal of this exercise is for the user to compile a TE library formed of TE consensus sequences corresponding to an organism of choice.

The set of guidelines described below recommends the use of many scripts and software applications. Instructions for software installation and detailed protocols describing each step of the analyses are included in Supplementary Methods. Whenever possible, alternative web-based tools are suggested and these do not require software installation, just internet access. For the more complex steps, such as the manual curation of multiple sequence alignments to generate a consensus, tutorial videos have been included and are available.

From this point on, it is assumed that the reader is familiar with the structure and organisation of the different TE orders (i.e. LINEs, LTRs, DNA transposons, and so on). If not, we recommend the consultation of a review article [14–17]. To identify the different TE orders, and potentially to further classify TEs to the superfamily level, it will be imperative to know what they look like! We recommend that Figure 1 from Wicker et al. [14] and Table 1 from Feschotte & Pritham [18] are used as “cheat sheets” and kept at hand during the manual curation process. It is worth noting at this point that many authors may propose different classification regimes for TEs. Although in this work we will not focus on the fine details of TE classification, we emphasise that the manual curation process can be affected by the type of TE being curated, defined primarily by structural characteristics. The reader is advised to focus on the identification of these structural characteristics (ORFs, long- or inverted terminal repeats, target site duplications among others) rather than relying solely on the nomenclature of homology hits. Ultimately, the manual curation process may render a different classification than the one originally assigned to a sequence by the de novo TE prediction tool. Armed with the accumulation of structural characteristics and possible nucleotide/protein homology to known TEs, the majority of TE sequences should yield to classification.

### Preparing your working environment

We will use a set of programs and display aids to facilitate the manual curation process. To this end, it is recommended that the following software is installed and checked before work starts.

- Cd-hit - unix [19]
- Blast+ − unix [20, 21]
- Bedtools - unix [22]
- Pfam_scan.pl plus HMM database - unix [23]
- Multiple sequence aligner, generally unix, such as MAFFT [24] or MUSCLE [25]
- HMMER - unix [26]
- An alignment viewer (generally GUI) such as Aliview [27] or BioEdit [28]
- A pairwise alignment tool (generally GUI) such as Gepard [29]
- EMBOSS package - unix [30]
- Ucsc-fasplit - unix [31]
- R - unix [32]

For advice on how to install and run these packages as well as links to a conda environment file for quick set-up, refer to the section “Software Installation” in Supplementary Methods.

Many of these applications have online servers that can be used instead of the stand-alone command-line version. Where these are not available, we do recommend alternative tools. These are discussed in Supplementary Methods.

For the purpose of illustrating the manual curation process with examples, we provide a set of files which include a dummy chromosome, a set of predicted, putative TE families for the same chromosome and some representative manually curated repeats from that genome. These files are downloaded as part of the Github repository that accompanies this manuscript (https://github.com/annaprotasio/TE_ManAnnot) and can also be accessed via the relevant sections in the Supplementary Methods. The reader can follow each step of the manual curation process presented here using the dummy examples and following the tutorial in Supplementary Methods. The tutorial contains protocols, one for each of the steps below, making the learning and/or practising of each step accessible and modular in nature. For the more complex steps of manual curation, video tutorials are also available.

### Understanding the RepeatModeler2 output

**Fig. 1:**
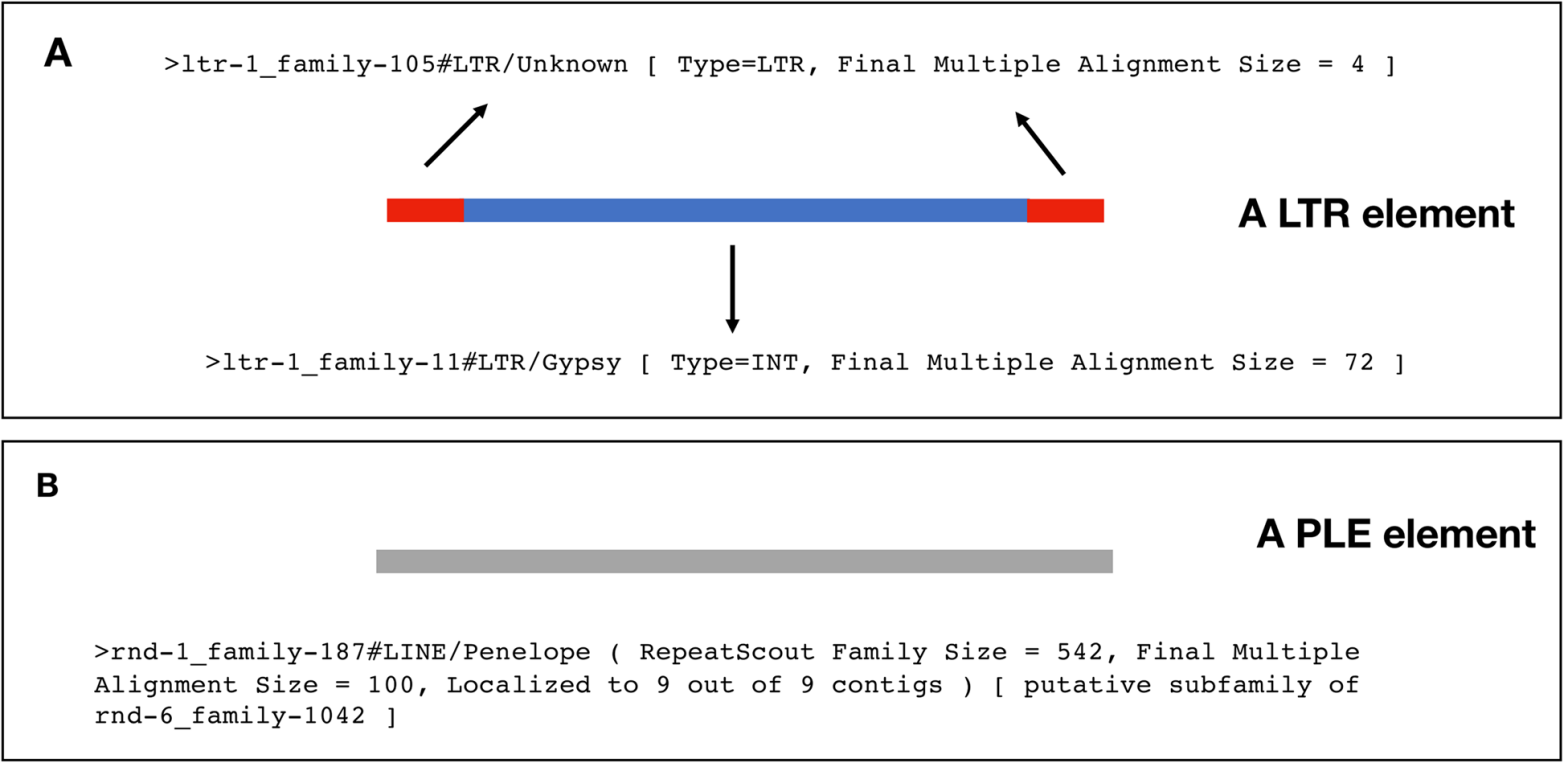
Schematic representation of RM2 output. **A** Sequences that are part of a LTR element. The internal and 5′/3′ LTR “subparts” are often presented as two different fasta entries / prospective families to allow for solo-LTRs (see solo-LTR in Other TEs). In the case of RepeatModeler2, the internal subpart is labelled “type = INT” (blue) and the LTR “Type = LTR” (red). In some cases “Type = LTR” can also refer to full-length LTR elements (combined red-blue-red blocks, as presented in the figure). RM2 will attempt to classify the family and this information can be found after the “#” symbol. In this example, the LTR subpart (red) is classified as Order LTR, superfamily Unknown and the internal subpart (blue) is classified also as Order LTR but superfamily “Gypsy”. When the LTR subpart and internal are reported in separate consensus sequences, it is possible to use manual curation to find the ones that form one full-length LTR element (see main text). The number after “Final Multiple Alignment Size” indicates how many sequences were used in the multiple sequence alignment to generate this consensus. **B** Sequence representing a prospective PLE predicted by Repeatmodeler2. The fasta header includes the classification, the software used for the prediction (RepeatScout), prospective family size and potential family relationships. Notice that the Family size (number of copies found in the genome) is different from the Final Multiple Alignment Size, which is limited to 100 to ease computation. Also note that PLEs are not LINEs (see Appendix I in Supplementary Methods), although for historic reasons they are classified as such by several databases and pipelines

As mentioned before, RepeatModeler2 (RM2) is one of the most popular tools for de novo detection of TE sequences in genomes. Because of its popularity, we will use the RM2 output as the starting point for these guidelines but, in general terms, the steps described below are applicable to the outputs of other de novo TE finders (with the exception of any scripts that refer to the specific output layout of RM2 such as extracting information from the fasta headers, see below). It is highly recommended that the reader has an understanding of the steps taken by the TE prediction tool used in their analysis. This will help understand the output. In the case of RM2, this pipeline combines various popular TE detection tools, the outputs of which are combined to provide a collection of putative TE families, from this point on called raw “RM2 families”. These are output in a multi-fasta file and each fasta header contains information about the putative TE family (Fig. 1). This information is based on the structural (only for LTR elements) and functional predictions made by RM2, based on sequence homology to known TE sequences and proteins. Therefore, the information that we can extract from the fasta headers (Protocol 1) needs to be taken in context with the tool used for the prediction. It is possible that different tools predict similar consensus families but classify them differently. Also note that the reliability of homology-based classification may differ markedly based on the existence (or not) of curated TEs from a species closely related to the target organism.

### Prioritising the output list

The output from RM2 can have thousands of potential TE consensus sequences and this makes it difficult to decide which ones should be prioritised for manual curation. In most cases, it is not practical to manually curate all of the sequences output by a TE de novo prediction tool. It is possible, however, to focus on a subset of sequences that are “most likely” TEs, that is to say, that have certain characteristics that resemble those of “real” TEs. We suggest using the following steps to produce a priority list of candidates. In brief, these steps will i) reduce the redundancy of the initial set of sequences since two highly similar sequences will eventually lead to the same curated consensus, ii) find the number of conserved protein domains (if any) encoded by each of the putative TE sequences; iii) calculate the length in nucleotides for each putative TE sequence, and iv) provide an approximate number of family members found in the genome. Detailed protocols describing how to perform each calculation are presented in the Supplementary Methods Protocols 2–6). A brief description of each is presented here:

- The first step is to reduce the number of sequences progressing to manual curation based on their sequence identity. To this end, we recommend using the program ‘cd-hit-est’ (Protocol 2). The output is a FASTA file with non-redundant sequences and a “cluster” with records of which sequences were clustered together.
- We can prioritise sequences based on the presence and number of protein domains (Protocol 3a and b). Autonomous TEs from most canonical orders/superfamilies have an expected set of proteins encoded in their sequence. We can predict conserved protein domains using profile-HMMs available from the Pfam database [23] and use this list to include or exclude sequences depending on the HMM match. For example, de novo TE predictions tools may include large families of closely related genes, typically recent expansions and gene duplications, that are not TEs but appear “as” TEs due to their copy number and similarity (i.e. repetitiveness). In these cases, sequences may have a non-TE Pfam HMM / or homology (blast) match and can be removed from the priority list.

The total number of TE-related domains per family can be calculated and used to sort the list of candidates. Alternatively, a ‘blastx’ homology-search (i.e. a nucleotide query against a protein sequence database) can be performed using either the NCBI web server (https://blast.ncbi.nlm.nih.gov) or on your local computer using the command line and a previously downloaded database (Protocol 3c).

iii) Another parameter for prioritising families for manual curation is the length of the prospective family (Protocol 4). In general, most organisms have TEs that range from a couple of hundred (e.g. SINEs) to many thousands of bases (~ 10–20 kb. e.g. Mavericks), with protein coding autonomous TEs in general longer than non-autonomous ones. We suggest that prospective families up to 10 kb should be prioritised for manual curation but longer families should not be ignored. The 10 kb suggested limit is a guideline that can make more sense when taken together with the number of copies found in the genome (see below).
iv) The number of copies in the genome can be another good tool to prioritise the prospective families, in particular when it is used in conjunction with other parameters such as the length of the family. It is possible to quickly perform a count of family members or best hits in the genome by running a homology search with the program ‘blastn’ [20] using the set of putative TE families as queries and the genome as a database (Protocol 5).

We recommend gathering all the information from the steps above into one table (Protocol 6). If the starting set of putative TE consensus families is the output of RM2, the script ‘generate_priority_list_from_RM2.sh’ available from the Github repository that accompanies this manuscript (see Supplementary Methods) can be used to produce a priority table (Protocol 0). The name of the putative TE families can be taken from the FASTA header, as well as the classification given by RM2 (LTR, LINE, DNA, etc) and whether the prospective family is a subfamily of another sequence (potentially these would have been removed with ‘cd-hit-est’, but this information can be useful in future steps). As mentioned in the Introduction, the classification extracted from the fasta header for each of the sequences may not be accurate and additional tools or user curation may provide a more reliable classification. The same applies to other parameters such as the number of conserved protein domains and length of the consensus. In our experience working with non-model organisms, sequence length and the number of conserved protein domains have a tendency to increase when comparing the RM2 consensus with the manually curated version.

In terms of prioritising which families should proceed into manual curation first, our recommendation is to start with those that:

- Show at least 1 TE-related conserved protein domain
- Their sequence length is within the limits of an expected TE family, that is approximately between 1 and 10 kb.
- Have at least 10 good quality ‘blast’ hits in the genome.

This is only a way of **prioritising** families and not a way of achieving a comprehensive set. If the process is limited only to those consensus sequences that meet the criteria above, relevant families such as those from SINEs (short sequences without a protein conserved domain) and other nonautonomous TEs will be excluded. However, the initial annotation of autonomous families often facilitates the identification of their non autonomous relatives, e.g. autonomous DNA transposons and their corresponding non-autonomous MITEs. This is because autonomous TEs and their non-autonomous counterparts often exhibit sequence homology over at least some of their length (e.g. at termini). Nonetheless, nonautonomous TEs can be targeted by relaxing the constraints on protein hits and consensus sequence length. The number of copies may also need to be reduced in species with smaller and less repetitive genomes. However, in our experience, this is a good place to start and many putative TE families that are redundant will be eliminated during the process of manual curation which is described below.

The quality and quantity of manually curated sequences will depend on how much manual work the curator is willing to put into achieving a final set. In principle, every predicted family could be checked and manually curated. However, is it sometimes not possible or practical to dedicate time and resources to this task.

### The manual curation process

**Fig. 2:**
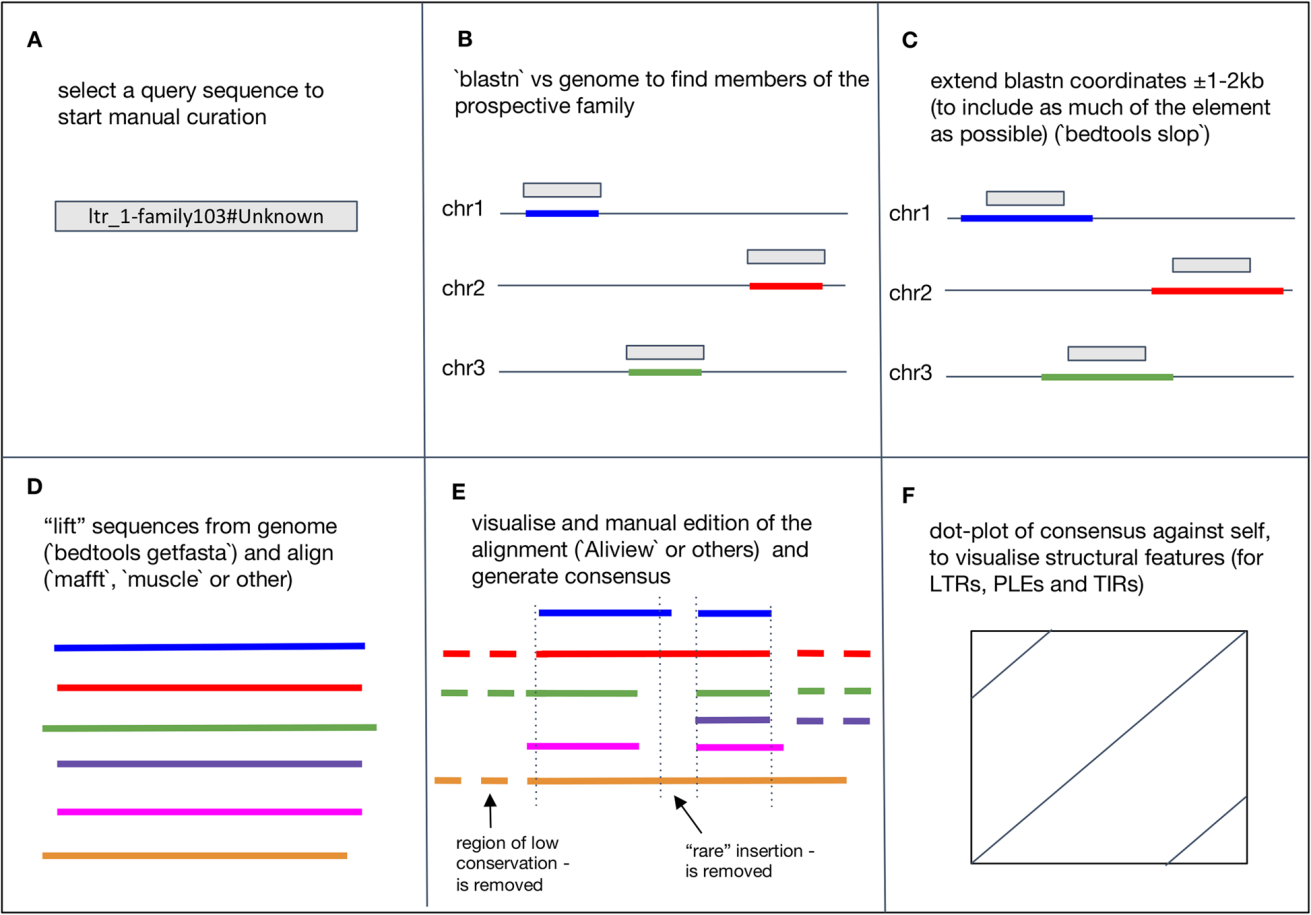
Overview of the process of manual curation. Graphical representation of the steps taken during the manual curation of a putative TE family. **A** A query sequence is selected to start the process. The selection can be based on the priorities that make it more likely to be an autonomous TE. **B** The putative TE is used as a query in a blast search against the reference genome and hits are recorded. **C** In most cases, the prospective families are a truncated version of the true TE, and thus the hit coordinates in the genome will also be truncated. To capture as much TE sequence as possible from the genomic location, the hit coordinates are extended by a given number of bases up- and down-stream in the genome. **D** The genomic sequences are extracted from the genome using ‘bedtools getfasta’, a multiple sequence alignment (MSA) is generated and written to a file. **E** The MSA is visualised in an alignment viewer and manually curated and a consensus is generated. **F** The final curated TE consensus is compared to itself to check for completeness. Using a dot-plot is particularly diagnostic in the case of elements flanked by repeats (as in the case of LTRs) but it is also possible to assess completeness by searching for conserved protein domains

The suggested steps for manual curation of a family are listed below. For a graphical overview of the process, see Fig. 2.

- Finding family members in the genome and alignment. Because the aim is to build a consensus sequence of a TE family, we have to collect as many members of this family as possible from the genome. One way of doing this is by performing a homology search with ‘blastn’ using an individual prospective TE sequence (e.g. one of the raw sequences from the RM2 output) as the “query” sequence against the genome “database” (see Protocol 7 on how to isolate a single sequence from a multi-fasta file). The results can be processed to collect the best hits and extract their nucleotide sequences from the genome using a tool such as ‘bedtools getfasta’. In most cases it is also advisable to extend the flanks of each genome hit to capture as much of the TE boundaries as possible (typically between 500-1500 nucleotides at either side), since the original model may not be complete. This can be achieved using the script ‘make_fasta_from_blast.sh’ provided in the Github repository that accompanies this manuscript and explained in the Supplementary Methods (Protocol 8 and Video 1). The output is a multi-FASTA file of all the putative copies of the query in the genome. The next step is to align the DNA sequences (Protocol 9). Multiple sequence alignment (MSA) is a critical step in generating high-quality TE libraries and consequently, careful attention should be paid to choosing the right tool. Our preferred alignment algorithm is MAFFT [24] because it is fast even with large files of 100–200 sequences as input and has been shown to perform well with low and medium divergence TE sequences [33]. If the ‘make_fasta_from_blast.sh’ script was used the sequences are already in the right orientation, otherwise you can add the option ‘--adjustdirection’ to MAFFT. The script ‘make_align_from_blast.sh’ incorporates the alignment step with MAFFT to ‘make_fasta_from_blast.sh’. Alternative multiple sequence alignment algorithms such as MUSCLE [25] can also be used. The resulting alignment file can be opened in Aliview [27] or your alignment viewer of choice. For full details on this step refer to Protocol 9. In the case of very large families, i.e. many hits in the genome, the input file for alignment may be too long (more than 100 sequences) for the purpose of visualisation. In this case, it is possible to create a subsample of the sequences collected from the genome. This process is described in Protocol 10.
- Alignment’s manual curation. In this section we discuss examples for LTRs, LINEs and DNA transposons. These are arguably the most widely known TE structures and are easily identified by de novo TE predictors. Other less well-known but equally important TEs are discussed in later sections of this manuscript. Screencasts showing the process of manual curation of alignments can be found in Videos 2 and 3.

**Fig. 3:**
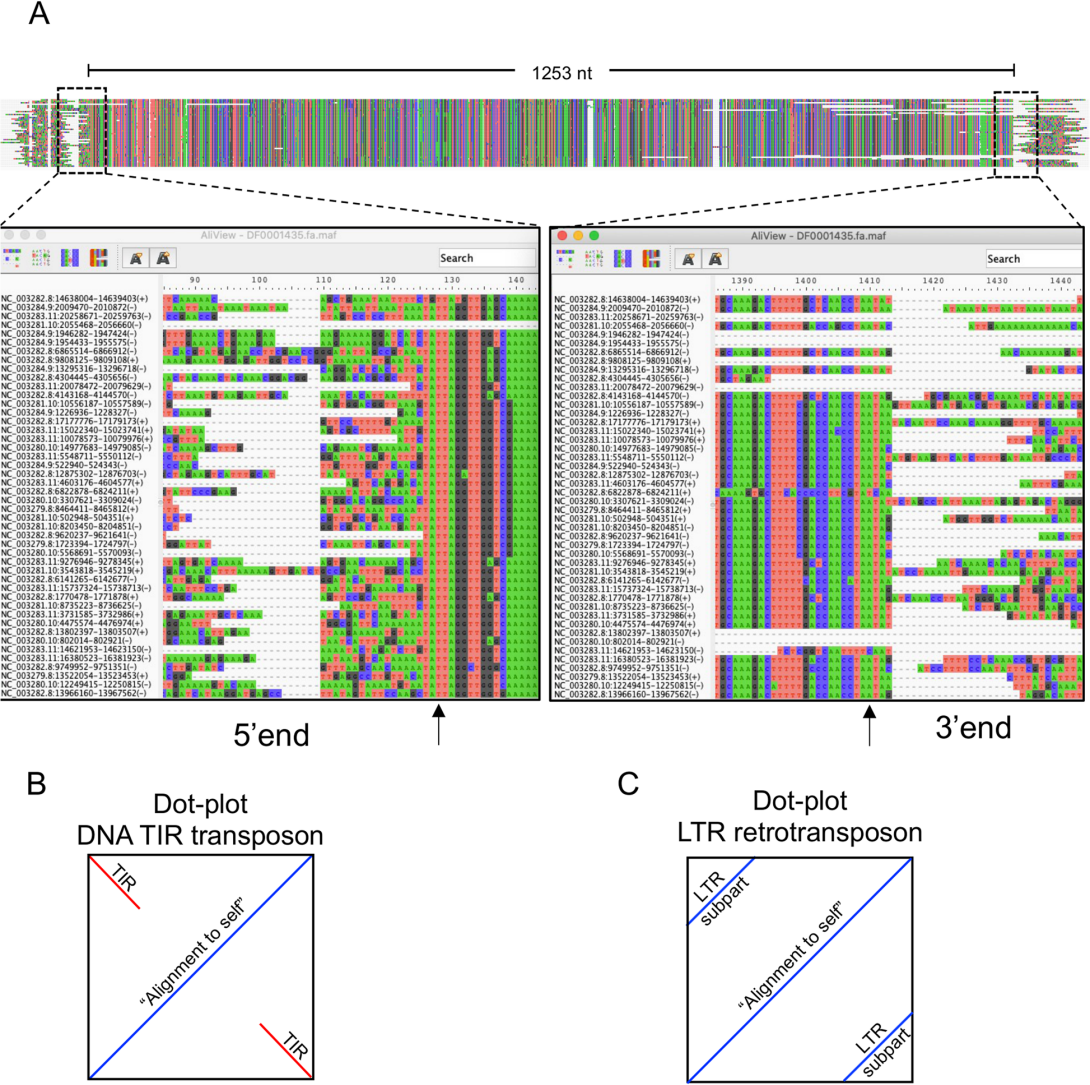
Visualisation of Class II DNA TIR Transposons and Class I LTR elements. **A** Multiple sequence alignment of Mariner3_CE family. The consensus sequence of *Caenorhabditis elegans* “Mariner3_CE” autonomous DNA transposon (Dfam accession DF0001435.1) was downloaded from Dfam [34] and used as query to search the *C.elegans* genome (accession GCF_000002985.6) for highly similar sequences - see Protocol 8 in Supplementary Methods. The MSA was generated with MUSCLE [25]. The resulting alignment is seen at the top, with dashed boxes marking the start (5’end) and end (3’end) of all family members. Bottom panels show respective magnified views of either end of the alignment. Arrows indicate first (5’end panel) and last (3’end panel) nucleotide of the alignment corresponding to start and end of the consensus sequence for DF0001435. Mariner DNA transposons show a TA dinucleotide TSD and this can be seen before and after the nucleotides indicated with the arrows. An MSA of full-length LTR element family has the same “shape” as for DNA TIR transposons, with two “blunt ends” of sequence similarity at either end of the alignment. **B** Class II DNA TIR transposon representative dot-plot. Terminal inverted repeats are shown in red and are perpendicular to the diagonal representing the self-alignment. The longer diagonal line represents matches between position 1 of sequence A and position 1 of sequence B, etc. For structural diagnostics of TEs, dot-plots are made entering the same sequence (i.e. the consensus TE) as sequence A and sequence B. **C** Class I LTR retrotransposon representative dot-plot. Long terminal repeats, sometimes referred to as “LTR subparts” and found at the 5′ and 3′ end of LTR elements, are shown in blue and are parallel to the diagonal showing the “self-alignment”. Contrary to TIRs in DNA transposons, where the sequence is inverted at either end of the TE, LTR subparts are terminal repeats with a tandem orientation

After opening the alignment in a visualisation tool such as AliView, the first step in the manual curation process is to identify the termini of the TE family. Because of their overall structure, some types of TEs have more structured boundaries, for example individual copies of LTRs and DNA transposons have defined 5′ and 3′ ends (direct and inverted repeats, respectively), providing consistent and easy-to-spot “blocks of homology” that make it easier for the curator to define the termini (Fig. 3). It is often useful to open two separate windows of the alignment viewer so the 5′ and 3′ ends can be viewed simultaneously. In the case of LTR families, these show canonical TG dinucleotides at the 5′ end and CA dinucleotides at the 3′ end. In addition, target site duplications (TSDs) can be generated during the transposition event immediately flanking the termini of the TE, and identifying TSDs can provide further support that the correct termini have been defined. Each family of LTRs or DNA transposons generally have a characteristic TSD length that can be highly informative when classifying TEs.

**Fig. 4:**
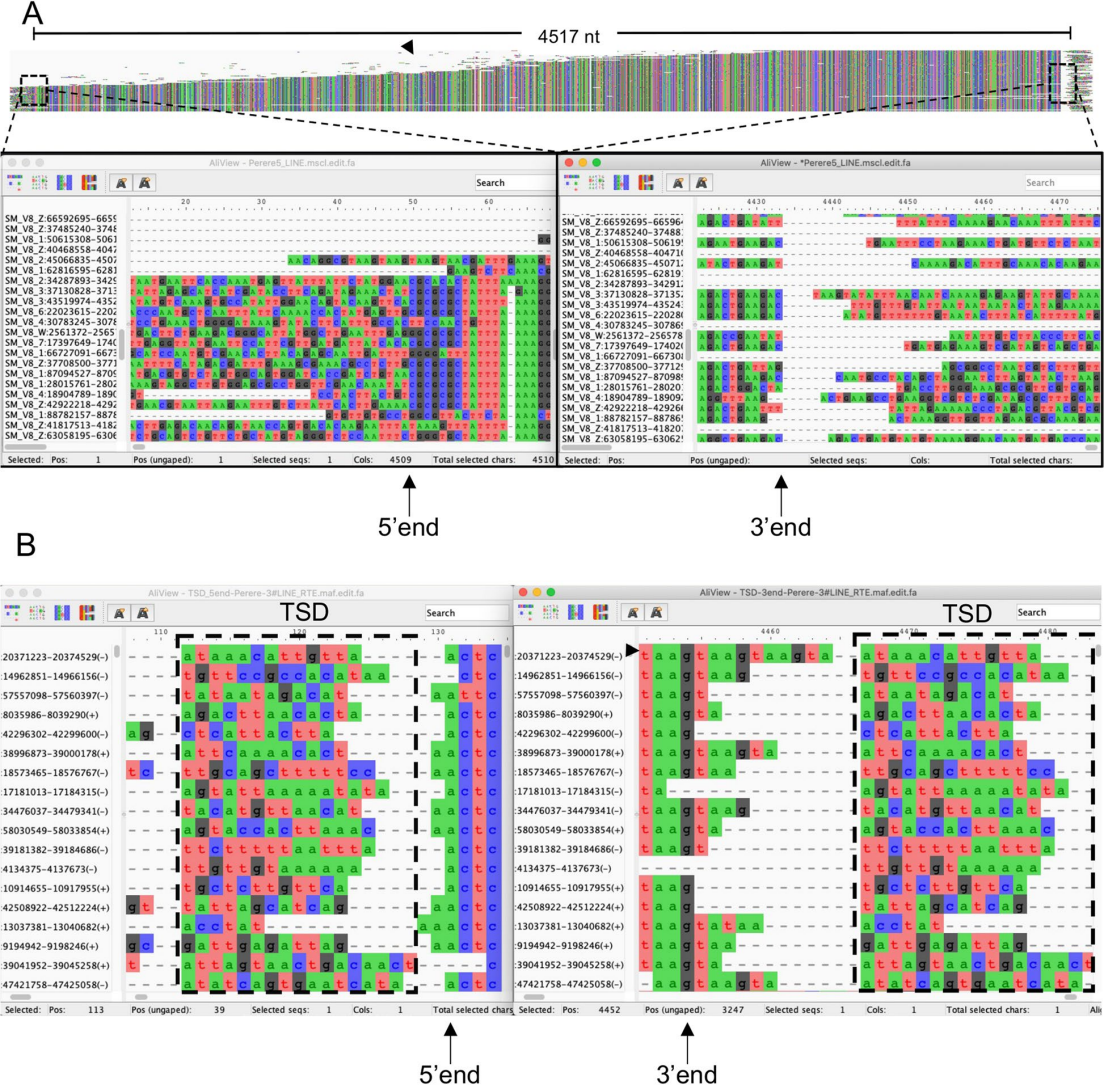
Visualisation of Class I LINEs. **A** Multiple sequence alignment (MSA) of a LINE retrotransposon showing typical shorter copies of the TE that manifest in truncations at the 5′ end. The consensus fasta sequence of *Schistosoma mansoni* “Perere-5” autonomous LINE retrotransposon (GenBank accession BN000796.1) was downloaded and used as query to search the *S. mansoni* genome for highly similar sequences (Protocol 8). The MSA was generated with MUSCLE [25] and re-ordered manually for display purposes. The resulting alignment is seen at the top, with dashed boxes marking the start (5′ end) and end (3′ end) of the alignment and the respective magnified views. Arrows indicate the first (5′ end panel) and last (3′ end panel) nucleotides of the alignment that correspond to the start and end of the TE alignment of the full-length consensus sequence. Some of the sequences that are part of this alignment are truncated at the 5′ end and are shown as shorter sequences in the alignments (arrowhead). In this particular example, a number of full-length sequences retrieved from the genome (as evidenced by high homology and sharp transition to non-homologous flanking sequences at the 5′ end of the alignments) enables generation of a full-length consensus sequence. **B** Target Site Duplications (TSDs) are found flaking TE insertions. A LINE retrotransposon is used to illustrate how TSDs can be seen in a MSA. Unlike the consistent TSD length of LTR and DNA transposon families, LINEs, as in the example, have variable length TSDs (dashed boxes). In this example, the 3′ end of the alignment is jagged due to the presence of a microsatellite GTAA (arrowhead indicates a sequence with two full copies). The bottom panel (in black and white) shows hypothetical TSDs that illustrate the variability observed in biological settings

A different case is presented by LINE elements. As a result of 5′ truncation upon insertion, copies of LINE families will show a range of sizes extending from the 3′ end towards the 5′ end exhibiting different degrees of completion (Fig. 4a). In these cases, it is best to concentrate on defining the 3′ terminus and search for the TSDs (Fig. 4b) to try to capture the whole of the element. The variable nucleotide length of LINE TSDs [35] make their identification a very laborious task, resulting in a small number of complete elements and biasing the consensus generation. An alternative approach is to capture as much aligned sequence as possible extending towards the 5′ (i.e. either until several copies terminate at the same point signifying the 5′ end of the complete element, or you are left with only two copies signifying that the element is incomplete). AT-rich microsatellites of variable copy number are often found near the 3′ end of LINEs. Once the termini have been determined (or 3′ terminus in the case of incomplete LINEs), blocks of the alignment that are not considered part of the TE family can be cropped/deleted using the tools in Aliview (see the Videos 2 and 3 for a visual guide).

An initial MSA may feature copies from more than one family (or sub-family). Because each prospective TE is used as an individual query in a BLAST search against the genome, hits may be retrieved for TE copies of a family closely related to that of the prospective TE, alongside copies from the family of interest. In such cases two (or more) sub-groups of sequences, which align far better to each other than to copies from the other sub-group, can be observed. Care should be taken to proceed only with the sequences belonging to a single family, while any other families present within the MSA can be returned to later (i.e. a single MSA may yield more than one consensus sequence if there are sufficient copies, although see tips from “sanity checkpoints” below to ensure that the same families are not mistakenly curated more than once!). In these cases, when visual inspection of the MSA results in two or more defined sub-groups, the curator can split the MSA, creating a new MSA per group (Video 4). The splitting of sub-groups or sub-families can also be done with a clustering approach (Protocol 11).

The next step is to clean the alignment by removing rare insertions (for example, when 1–2 sequences in a total of 30 have an insertion) and highly divergent sequences or sections of the alignment. The multiple sequence alignment tool ‘t-coffee’ [36] offers, in addition to its alignment capabilities, the possibility of removing alignment “gaps” that are the result of rare insertions. This tool proves extremely useful when manually curating a large number of alignments (usage of ‘t-coffee’ is described in Protocol 9). Alternatively, if the tool used to produce the consensus sequence is capable of recognising gap characters (i.e. a gap can be the consensus “nucleotide”), rare insertions can safely be kept and their corresponding gaps can later be deleted from the consensus sequence.

While performing gap removal, splitting of the MSA into sub-families or any MSA manual curation step, the sequences can be realigned at any time and the process repeated until a set of well-aligned sequences is obtained. In fact, iteration is often required for very long TEs, where an initial extension of flanking regions of 1–2 kb is insufficient to capture the full TE.

There are certain characteristics of some TE families or their individual copies that can make curation difficult. On many occasions, and especially for inactive TE families, the genomic copies can be quite fragmented making the task of annotating the full element difficult. It is possible to use a highly abundant yet partial sequence or fragment as the consensus for the library. However, it is best to use one or two of the longest representatives to check if the element contains additional protein domains or identifiable termini. This is true for many classes of TE, such as LINEs, LTRs and DNA/TIRs (see section on “Special cases”). Another example is found in TEs that harbour satellite or microsatellite repeats (e.g. in some LINEs [37]). In this case, the tandemly repeated regions in the different TE copies may have variable lengths, complicating the alignment. Another common issue is the non-independence of retrieved copies. This can occur if a section of DNA containing the TE of interest has been tandemly duplicated, or if there are errors in the genome assembly that cause redundant sequences. These cases can often be identified by the alignment between two or more copies extending beyond the termini of the other copies. In such cases all but one copy should be removed to avoid biasing the consensus sequence.

This last section focused on the manual curation of a sequence alignment. As its name clearly indicates, this is a manual process and therefore highly subjective to human error, especially for older TE families with highly divergent copies. Tools such as ‘t-coffee’ (mentioned above), ‘trimAI’ [38] and ‘CIAlign’ [39] (see section Automation) can be extremely useful in adding reproducibility to the process of removing insertions/deletions or trimming alignments based on differences in sequence conservation. Therefore, it is highly recommended that an automated method is applied whenever possible. However, if the results obtained from automatic curation do not reflect the curator’s expectations of how the final MSA should look like, it is always possible to return to manual curation.

iii) The next step is to produce a consensus sequence from the alignment. The function ‘cons’ from the EMBOSS package produces a consensus sequence from an MSA (Protocol 12). Applied with default parameters, this tool is quite conservative and avoids making a decision when divergence at a particular site is high. This results in many Ns in the output. The parameters in ‘cons’ can be adjusted to avoid this situation, forcing it to make a decision based on the available information (see program help ‘cons -help’ for more details). Other tools include ‘t-coffee’, that provides a consensus generating function that can be run separately or as part of the gap-removal step, or the web-based tool “Advanced Consensus Maker” [40].

Regardless of which tool and parameters are used, we highly recommend that consistency is kept during the whole process of manual curation so all consensus sequences are produced in the same manner. It is generally recommended to check the consensus against the alignment in the sequence viewer of choice, to quickly confirm that no remaining poorly aligned sequence has been incorporated.

Although generating a consensus sequence from a MSA has some advantages (see next sections), it is arguably a process that fails to record the sequence variation present in members of the family. This drawback is overcome by using profile-HMMs [34] that define a TE family in a similar way that a consensus sequence can albeit with much more information because an HMM can capture the nucleotide variation in each position. However, the use of consensus sequences may still be preferred in downstream analyses such as the prediction of protein domains and the characterisation of structural properties present in a sequence. In our view, there is no reason not to produce an HMM (instruction on how to produce HMMs can be found in Protocol 12) *and* a consensus sequence at the same time, keeping both in record and using them when required in bespoke downstream steps.

iv) We can further check the consensus sequence for the presence of regions encoding conserved domains (Protocol 3) by extracting all possible open reading frames with the ‘getorf’ function from EMBOSS and running ‘pfam_scan.pl’ on these amino acid sequences (Protocol 3a), or alternatively directly running ‘blastx’ using the consensus sequence in DNA form against a pre-collected database of known TE proteins (Protocol 3b). While generating a conceptual translation using ‘getorf’ it is possible that no domains are found. There could be two primary reasons: that the consensus sequence does not encode proteins, as is the case for most non-autonomous TEs, or that there are stop codons, frameshifts or introns that prevent the generation of a viable ORF. If the second option is suspected, it is recommended to instead produce a conceptual translation of the DNA sequence using the ‘transeq’ function (also from EMBOSS) including the ‘-clean’ option. This will change stop codons into Xs that can be ignored by Pfam while producing longer peptides.

Most TEs have characteristic protein domains that facilitate their classification. Furthermore, the order of protein domains and the presence/absence of additional domains can be useful for further classification to the superfamily level. For autonomous LTR elements, these conserved domains can be found in one and sometimes two ORFs and encode a N-terminus “GAG” structural protein followed by a “POL” enzyme. The “POL” protein is in turn formed by several domains: an aspartic protease, a reverse transcriptase, RNAse H and an integrase domain. This is the general organisation of all LTR superfamilies, although in *Copia* elements the domain order differs and in endogenous retroviruses an additional ENV domain is present at the C-terminus. Autonomous LINEs also encode “POL” proteins containing endonuclease and reverse transcriptase domains. The reverse transcriptase domain may be located just before the C-terminal (RTE, Jockey, L1), separated from the C-terminal by an RNAse H domain (superclass I) or be found at the N-terminal (R2 superfamily). An additional N-terminal ORF1 protein is found in Jockey, L1 and I superfamilies. The most well-described DNA transposons (Class II) encode a DD[E/D] transposase and, depending on the superfamily, may or may not be accompanied by a second ORF. The DD[E/D] denomination refers to the amino acid triad that participated in the catalytic pocket (reviewed in [41]). Therefore, the presence of a DD[E/D] domain and structural TIRs in a consensus sequence is a very good indicator of a DNA transposon.

The different protein domains characteristic of each TE order are reviewed and summarised elsewhere [14, 16, 17, 41–43]. In addition, an overview of the less known TEs can be found below in the section “Other TEs”.

**Fig. 5:**
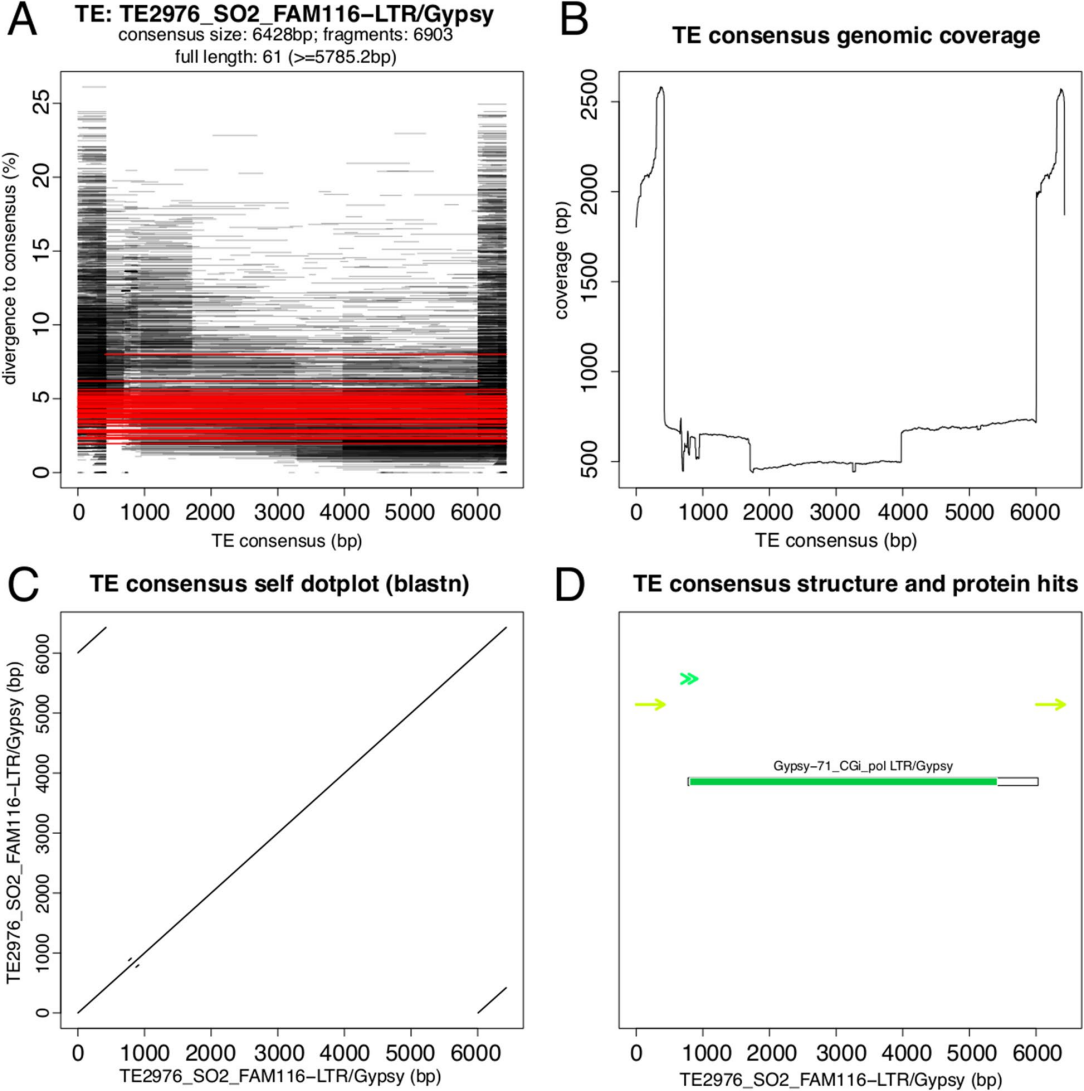
Example of TE-Aid output. TE-AID produces a set of plots and graphical representations that helps understanding the nature of the query sequence. This example features the consensus sequence of a LTR/Gypsy element of the rice weevil *Sitophilus oryzae*. In all plots, the horizontal axis represents the length of the query sequence (6000 nt in this example). **A** Fragment and divergence plot. Each blastn hit retrieved from the genome is represented with a line. Red lines are hits regarded as “full-length” and by default > 90% of the query sequence. Location on the y axis represents the % divergence with respect to the query (the higher the divergence the more base changes there are); by default hits are plotted with some degree of transparency to identify highly represented fragments. **B** Coverage plot representing the depth of coverage from blastn hits for each section of the query. A height of 2500 over the first 500 bases of the 5’end indicates that on average ~ 2500 copies are found in the genome whose sequences are homologous to this section of the query. In this example, there are many more blastn hits of the LTR subparts than for the internal part of the LTRs, likely representing the high frequency of solo-LTRs in the genome. **C** Self-alignment dot-plot reveals repeated sequences in the query - see Protocol 14 for a full explanation. The LTR subparts can be identified as parallel lines in the top left and bottom right of the graph. **D** Graphical interpretation of the dot-plot (top) and representation of open reading frames (black lines) and structural TE protein hits. The LTR subparts are shown as light green arrows. The arrowheads (“> >”) in dark green represent micro-homology along the sequence, though not diagnostic for this family of TE. A single open reading frame (black box) has been found and the translated protein is homologous to a known LTR/Gypsy polyprotein (POL) gene (green box)

e) The next step is to confirm the structural integrity/characteristics of the consensus. To facilitate this process, we have developed the tool ‘TE-Aid’ which produces a set of plots and graphical representations that helps TE classification (Protocol 13). TE-Aid can also provide information about the completeness of the TE consensus based on, for example, presence of both LTR subparts in LTR elements and/or presence of conserved protein domains. The plots produced are a “coverage plus divergence” plot for events in the genome that match the consensus (query), a representation of the blastn hits found in the genome, a self-alignment dot-plot (Protocol 14) and a graphical representation of internal repeats and ORFs (Fig. 5). This set of diagnostic images can be run at the beginning and/or end of the manual curation process. Running TE-Aid at the start may help identify raw repeat models that may already be entirely or almost full-length. See Supplementary Text file for a comprehensive image gallery of typical TE-Aid outputs.

f) Once a curated TE consensus has been generated and checked, the sequence can be used to search known TE families to find out if the same TE or TE family has been previously identified. Databases such as Repbase [44], RepetDB [45] and Dfam [34], amongst others (a comprehensive list of databases can be found in TEhub.org), provide repositories of known TE sequences and can be queried against prospective new TEs. If a match is found (based on the 80–80-80 rule, see definition of “TE family” in Glossary box), it is highly recommended that name and classification are the same between both sequences. If no matches are found, the newly curated TE family is a new family and a new name will have to be assigned.
g) The final step is naming a newly curated consensus sequence. Early examples of TEs were regularly given independent creative names, for example in relation to jumping/travelling (*Gulliver, Pioneer, kangaroo* etc.) or figures from mythology (*Helena, Penelope,* etc.). However, with thousands of genome projects now underway, and every species expected to harbour tens or hundreds of TE families, contemporary TE naming requires a more systematic approach. One method, similar to that used by the TE repository Repbase [44] is to use the format “superfamily-X_yYyy”, where X is a unique number and yYyy is a four-letter identifier for the species in question. For example, two *Gypsy* LTR elements from the genome of the spike moss *Selaginella moellendorffii* could be named *Gypsy-1_sMoe* and *Gypsy-2_sMoe*, respectively. If subfamilies have been curated (Protocol 11), these can be specified by letters following the number (e.g. *Gypsy-1a_sMoe*, *Gypsy-1b_sMoe*), and nonautonomous elements can be specified with an “N” preceding the number (e.g. *Gypsy-N1-sMoe*). If the superfamily is not known the order can be used (e.g. *LTR-1_sMoe*), and in cases where superfamilies have been clearly divided into subclades (as is the case for plant LTRs [46]) it may be more informative to use the subclade name. Another proposed method has been presented by Wicker et al. [14]. In their naming system the authors suggest a 3-letter abbreviation for each TE superfamily followed by the family name assigned by the curator followed by the accession number of the sequence in which a given copy is found. For example, RIL_L1HS_NC_000001–7 is a Class I element of order LINE, superfamily L1, found in the chromosome/DNA sequence with accession number NC_000001, and this repeat is the 7th annotation of that given family. This method does not include the species in the name, although this information is easily found using the accession number in any public database. Due to the inclusion of the accession number, this last method allows for the exact identification of the sequence where the TE was found including genome version for those organisms that have multiple genome assemblies available. In conclusion, although numerous naming approaches exist, we encourage annotators to use systematic and easily catalogued approaches.

The exact order of the steps mentioned above is not critical and they can be changed, omitted or added to meet the needs of the curator. For instance, for very old and inactive TEs where the family members show substantial divergence, it may not be practical to attempt to find the exact boundaries of the consensus, and perhaps it is more important for the curator to find clues that can inform TE classification based on sequence homology of any remaining stretches of amino acid sequences. In this case, the curator may put more effort into predicting the conceptual translation of any remaining ORF without paying much attention to achieving defined boundaries of the alignment of members of the TE family. Depending on the goal of this annotation practice, it may not be effective to dedicate much time to such “dead” TEs.

A consolidated TE library is saved as a multi-fasta file where each entry contains the consensus of a manually annotated TE. Non-curated TE sequences can also be included but it is advisable to add the suffix ‘inc’ to their file name, indicating that these are incomplete. Downstream analyses may or may not include the incomplete TE annotations. In the vast majority of cases, the immediate following step is to use RepeatMasker [47] to find and annotate all TE copies in the genome. It is often the case that LTR subparts are entered separately in the TE library, that is to say, one fasta sequence for the LTR subpart and one for the internal subpart (no second LTR subpart is entered as the 5′ and 3′ end are the near-identical). This organisation of the TE library is the one followed by Repbase [44] and may facilitate the localisation of solo-LTRs (see below) in genomes and the use of additional annotation tools [48]. Submission of a consolidated TE library is highly encouraged and can be done to public repositories (for example, Dfam [34]) through which other researchers will be able to access this valuable data.

### Automation

An alternative to manually curating the MSA is to use the recently created tool CIAlign [39]. CIAlign removes both indels appearing in the minority of sequences and highly divergent and short sequences, building a consensus sequence at the end of the process. Application of CIAlign can be automated and applied to many alignments at the same time. Further curation of the alignments should be much easier and straightforward after CIAlign has been applied. A bespoke application for the semi-automatic curation of TE alignments would be very beneficial for the process of manual curation, not only making the process quicker and less labour intensive, but providing more reproducible results.

Trimming low conservation edges of the alignments can also be automated using the algorithm ‘trimAl’ [38]. However, for the new curator, we recommend visual inspection of the alignments to become familiar with the structures that these can present, and we find visualisation essential to perform troubleshooting. In summary, implementation of ‘CIAlign’ and ‘trimAI’ could dramatically reduce the time and effort spent in manual curation. Although these tools have not been designed with TEs in mind, it is possible to define a set of parameters that would fit the requirements of the TE manual curation. It is beyond the scope of this work to define such parameters and we therefore encourage the curator to experiment with their application to the manual curation process.

Many of the steps outlined as part of the manual curation process can be automated so only one command is needed to run the same set of processes on all the prospective TE sequences. This is particularly the case for command-line programs (such as the use of ‘cons’, ‘getorf’, etc) and is achieved by running a command using a loop (Protocol 15).

### Sanity checkpoints

Because the list of potential families is likely to be quite vast, it is a good idea to incorporate some checkpoints that can be used periodically to assess progress, reducing the amount of time that is spent on curating the same families without noticing (believe it or not, it happens frequently). One possible checkpoint is to evaluate the set of manually curated families against the raw RM2 output. As we have described for the manual curation process, TE copies are found in the genome and the loci coordinates extended up- and down- stream to include as much TE as possible. During this process, we sometimes inadvertently include putative families that were predicted by RM2 but not “joined” in the RM2 family output. By running a ‘cd-hit-est’ (or a stringent blastn) on the combined set of curated and raw RM2 families it is possible to detect any raw RM2 families that are now contained in the manually curated set. Consequently, these can be removed or flagged from our table and considered as “clustered” or already represented in the manually curated set. This scenario is particularly important for LTR elements because in many cases RM2 will output the internal (named type = “INT” in the RM2 output) and the LTR subarts (type = “LTR”, typically short sequences in the order of 200-600 nt) separately. During the process of manual curation and when starting from an internal sequence, we will without doubt find the LTR subparts and include them as part of the LTR element. If we fail to include the checkpoint, we run the risk of starting another round of manual curation from the LTR subpart or another subfamily of the same family, extending and ending up with the same consensus, having spent double the necessary time to curate the same family! The same logic applies when two families may have been captured and curated from a single raw RM2 sequence and MSA (see Section 4b - Alignment’s manual curation), since the “extra” family may be present as its own raw RM2 sequence that could be curated a second time if it were not recognised and filtered out.

### Other TEs

In the previous sections, we addressed how to manually curate TEs in general, using examples from the most widely known TEs, namely autonomous LTRs, LINEs and DNA transposons. However, there are several other types of TEs and their structural differences have a consequence in how we manage their annotation. Many of these TEs are increasingly receiving more attention due to their identification in a wider set of host species. In the following lines we briefly describe some of the key features of these TEs, and additionally describe the special case of solo-LTRs. Examples of all TE orders and more detailed information on their structures, organisation and protein coding capacities are provided in the TE-Aid gallery, and further notes on other TE orders can be found in the Supplementary Methods’ Appendix.

***Penelope*-like elements** (PLEs) are an enigmatic order of retrotransposons (Class I) that are evolutionarily distinct from LINEs and LTRs [49, 50]. PLEs are 5′ truncated upon insertion, and on first viewing they may superficially resemble a LINE. However, full-length insertions are immediately followed by a second copy inserted in tandem. This second copy is itself usually 5′ truncated, producing a structure called a pseudo-LTR (pLTR), which as the name suggests resembles an LTR. Other specific peculiarities can be observed in individual copies, including inverted pLTRs and short extensions at the 3′ end (see figure 2 in [43]). A “complete” consensus sequence will extend from the 3′ terminus to the sequence immediately preceding the pLTR (since this structure is formed by a second copy it is not necessary to include in the consensus).

**DIRS** are another distinct group of retrotransposons, which can be identified from initial alignments by their unique termini and lack of TSDs. Two distinct types of DIRS exist (reviewed in [42]). The first has inverted terminal repeats (ITRs), which can be imperfect, and a short internal complementary region (ICR) located at the 3′ of the internal region. The 5′ of the ICR is complementary to the beginning of the 5′ ITR, while the 3′ is complementary to the end of the 3′ ITR. The second type is characterised by split direct repeats (SDRs), where there are two pairs of direct terminal repeats termed A1 and A2, and B1 and B2. The most common structural representation is “A1-internal-B1-A2-B2”, although other configurations exist. Given their distinct termini, curating DIRS families will generally be a similar experience to LTRs and DNA transposons (see above).

**Helitrons** are DNA elements (Class II) with relatively inconspicuous structural motifs and generally no TSDs, so determining termini can be very difficult. It is recommended to use a partial sequence that covers the internal protein coding region unless many copies exist and the termini can be confidently defined. If termini have been determined, two distinct structures have been described (reviewed by [51]). Canonical Helitrons contain a single hairpin structure at the 3′, are characterised by 5′ TC and CTRR 3′ ends, and insert between A and T nucleotides. The second type contains hairpins at each end and short asymmetrical terminal inverted repeats (ATIRs), and are further characterised by a 5′ T terminal nucleotide and insertion upstream of a T. In both cases the hairpin and ATIR structures may be too small or imperfect to show up in dot plots, making their characterisation challenging.

**Cryptons** are another order of enigmatic DNA elements [52, 53]. They can generally be identified based on their coding sequence (see TE-Aid gallery), with the only distinguishing feature a short (several bp) direct repeat at the termini. This will resemble a TSD, although the sequence will be similar across all copies. Cryptons are generally low copy number, and again achieving a consensus that represents the internal protein coding region may be sufficient.

**Mavericks/Polintons** are the final described eukaryotic order of DNA elements [54, 55]. These elements may resemble giant (15–40 kb) DNA transposons, in that they contain long TIRs (hundreds of nucleotides) and feature 6 bp TSDs, although autonomous families can be easily distinguished by their unique protein-coding capacity (see references).

The above notes described autonomous TEs, that is to say, those TEs that encode the necessary machinery to execute their own transposition. However, many autonomous TE families have non-autonomous counterparts. These non-autonomous TEs typically lack one or many of the coding sequences that are necessary to produce the transposition machinery. Nonetheless, these elements are still able to transpose by using the machinery of their autonomous counterparts. For instance, a non-autonomous LTR element lacking the reverse transcriptase domain can still be transcribed and use the retrotranscriptase of an autonomous LTR element, after all, these enzymes are all being produced and are active in the cell cytoplasm. Here we describe some of the better known non-autonomous TEs.

Class I TEs, those that use an RNA intermediate during their transposition, can generate a wide range of non-autonomous versions of themselves. In the case of LTRs, non-autonomous LTRs can include some, most or almost none of the internal sequence. This could have been rendered non-coding due to accumulation of mutations but it is also common to see complete deletions of internal regions [56]. In some cases the autonomous TE can be very hard to find, either because it’s in low copy number or mostly degraded. Superfamily-level classification of non-autonomous LTRs relies on sequence homology to the autonomous counterpart since the sequence conservation in the LTR subparts are critical for the recognition of the transposition machinery.

**MITEs** (Miniature Interspersed Transposable Elements) are the non-autonomous version of TIR-DNA transposons. As mentioned earlier, TIRs (Terminal Inverted Repeats) are DNA (class II) transposons characterized by the presence of a transposase in autonomous copies, flanked by non-coding inverted repeats which serve as DNA-binding motifs for the transposase. Full-length DNA/TIR size can be very variable, with most known families in the 2 kb range, though some families may be > 10 kb in some species. Their length includes inverted repeats ranging from from a few bp up to > 1 kb. Through time, TIRs accumulate internal deletions leading to the loss of the transposase and their autonomy. When the coding potential is lost rendering the TE non-autonomous, the element can be categorized as MITE. MITEs typically are short, and range from 0.05-1 kb in length. In some cases, the complete internal part can be absent, leaving only the TIRs behind. MITEs can be mobilized by the transposase of their autonomous counterparts, often leading to the rapid accumulation of hundreds to thousands of MITE copies [57]. The presence of flanking inverted repeats is an easy diagnostic on a self dotplot. The presence of DD[E/D] transposase and a complete ORF will characterise an autonomous TIR transposon, while incomplete or broken up ORFs will indicate that the family is most likely represented by non-autonomous copies. However, degraded or incomplete ORFs should not be mistaken for transposase genes with introns, which are plentiful in some species/TE families [58]. For a given TIR family, a gradient from autonomous TEs to MITEs can be observed when the complete consensus is queried against the genome.

**SINEs** are small 80–500 nt long (but typically 150–200 nt) non-autonomous sequences that use a tRNA or 5S-rRNA gene as a transcriptional initiator and rely on the transposition machinery of LINEs to replicate. They use Pol III for transcription and therefore contain the Pol III promoter (TGGCNNAGTGGN_30–35_GGTTCGANNCC) in their sequence, approximately 10–15 nt from the 5′ end [59]. As is the case with many other TEs, transposition generates TSDs and the presence of TSDs should be used to confirm if any prospective SINE is real or not. Very often putative TE families in the RM2 output that are called “SINEs” or “Unknown” with hits to tRNA or the 5S-RNA have no additional sequence other than this match and are likely to represent just the tRNA or rRNA. One possibility to filter these out is using tRNA scanner [60] to check which of the “SINEs” or short “Unknown” families are identified as tRNAs. A similar approach can be used to find those that are rRNA genes by doing a Blast search against GenBank. If the set of tRNA and rRNA genes are known for the genome of interest, an alternative would be to use these sequences to mask the genome prior to running RM2 or to use them in a Blast search against all the prospective families at the start of the process. Finally, interested readers are directed to recent work describing metazoan retrozyme non-autonomous elements, which may rely on PLEs for their mobility and can be considered a new type of SINE depending on the definition used [61, 62].

**Solo LTRs** are generated when LTR elements loop out during an event of non-allelic homologous recombination involving both LTRs. This process leaves behind just one of the LTR subparts [63]. When this happens the target site duplication (TSD) sequences immediately up- and down-stream of the LTR will be intact. Many of the short (between 50 nt and 500 nt) RM2 output families classified as “Unknown” may be solo LTRs, and in some cases the full element may not exist in the genome. If these unknown families have 4–6 bp TSDs and start with TGT and end with CAC it can safely be annotated as a solo LTR. Sometimes variants of these ends are observed, eg. TGA and TCA, which can also be classified as solo LTRs. LTR subparts (those at the 5′ and 3′ ends of an LTR element) are often reported separately from the full-length LTR element to allow for the identification of solo LTRs in downstream analyses.

**Satellites** are typically short and highly repetitive DNA sequencing. Sometimes de novo prediction tools and/or pipelines output prospective families that are in fact DNA satellites. These are often classified under the “Unknown” label because they neither match a TE-related protein domain nor have a classifiable structural motif (such as LTR subparts). Satellites can be identified when the sequence is visualised using a self-dotplot as having many self alignments and usually no clear termini and no TSDs (Supplementary figure). If it is of interest to describe the satellite in more detail, Tandem Repeats Finder [64] can be used to define the monomeric repeat. Alternatively, it is possible to perform a satellite scan of the genome using, for example, RepeatExplorer [65] to identify these elements and, as with tRNAs and rRNAs, use the output to mask the genome before using it as input for the de novo TE finder. However, as previously mentioned TEs themselves can harbour tandem repeats (often microsatellites, but sometimes repeats with longer monomers), so care should be taken if such cases are encountered.

#### Other unknown repeats

As mentioned above, unknown repeats are those for which there is no recognisable TE signature, either a protein domain or a structural repeat. Many genomic repeats may end up in this category and they may or may not be relevant to the annotation of genomic repeat regions, depending on the objective of the analyses. In some cases, TE de novo algorithms can predict a large number of families with “Unknown” classification that are indeed fractions of larger better defined TE families or can also be highly degraded versions of the same. In this case, as progress is made in the completion of the TE library, it is prudent to run a clustering step with all manually curated and predicted sequences to investigate if the manually curated ones do include any of the putative TEs predicted as “Unknown”, or indeed others with an assigned classification. In our experience, this step tends to dramatically reduce the number of sequences classified as “Unknown”. Finally, some Unknown sequences may be multi-copy protein-coding gene families, which should be removed, or indeed even novel TE families, which could be studied further.

### Classifying the manually curated TE library

As alluded to in this work, TE families are often classified as part of superfamilies, major ancient clades within each TE order. These are defined by a combination of structural features, the type of proteins they encode for, and in some cases are based only on phylogenetic analysis. Various classification strategies exist [14, 66, 67] but it is outside the scope of this work to explore them. In the first part of these guidelines, we showed how RM2 includes a classification for each prospective family (Fig. 1). It uses a tool called “*RepeatClassifier*” that produces the automatic annotation of the prospective families based on their similarity to a set of curated TE proteins that are included with RM2 distribution. “*RepeatClassifier*” can be run independently on any TE library including one made manually (see the RepeatModeler2 Github https://github.com/Dfam-consortium/RepeatModeler/blob/master/RepeatClassifier). However, once again caution is urged if running this on species that are highly divergent (i.e. 100 s of millions of years) from any other species with curated TE libraries. Other applications include TEClass [68], which uses a machine learning support vector approach, and DeepTE [69], which uses convolutional neural networks.

### Community resources

With the arrival of more and higher quality genome assemblies it is now possible to more easily investigate the TE content of many organisms. Hence, the research community surrounding TE biology and tool development for their study has grown dramatically in the last decade. This friendly and welcoming community has a number of portals for exchanging ideas, methods and other TE tools. Of note, the recently established TEhub [12] is a community led “hub” for the centralisation of TE resources and Dfam [34] is a free and open access database of TE sequences, alignments and genome annotations. Another community tool is the Slack workspace “transposonsworldwide.slack.com”, with more than 500 members at the time of writing, open to all and with many channels tailored to the different discussion topics.

## Conclusions

In this manuscript we have presented one of many available ways in which it is possible to produce a manually curated library or sequence. These steps can be applied individually to one sequence of interest or to an entire set of TE sequence predictions as output by many TE prediction algorithms. It is therefore timely to briefly discuss the product of this very laborious task.

A consensus sequence derived from the multiple alignment and curation of the many copies found in a genome can be taken as the average sequence of the TE family. Though often used as a proxy of the ancestral TE sequence, it is critical to distinguish this consensus (made with a majority rule) from an ad-hoc reconstruction of the ancestral sequence, which would require the use of phylogenetic methods and hypotheses on the biology of distinct TE families [67, 70]. Although it is technically always possible to obtain a consensus from an alignment, there are certain situations where it may be more appropriate to choose the “best” possible sequence. For example, in the case of highly degraded (i.e. old, not actively jumping) LINE families for which most of the family members are truncated, it may be possible to choose the most complete sequence under the criteria that it contains as much of the coding sequence as possible. If all the sequences are truncated, it is also possible to build a consensus sequence out of the individual truncated sequences.

The process of producing a high quality TE library for any given genome is a never ending task and the curator may have to make decisions about modifying the work done so far. Even after days, weeks and months of manual curation it is often tempting to return to the library to edit another alignment, to include a family that somehow escaped through the net, to merge two or more families that share more similarities than originally thought, and so on. It is for the curator to evaluate how much more work should go into producing an even better TE library and this decision will rest on the purpose of the downstream analyses in mind.

The process described here as manual curation of TEs is just one view presented by the authors and it is without a doubt one among many. Each laboratory and each “TE expert” will have their own way of curating TEs and in fact, there might be as many methods as TE labs in the research community. Perhaps due to this variety, there has never been a set of recommendations or guidelines published so far. Lack of such resources within the literature can prevent researchers from outside the TE community from accessing this knowledge. The aim of this manuscript is to bridge the gap and provide a starting point.

We would like to emphasise that the tools presented here are just a few of the many excellent tools available for sequence analysis, some of them developed with TE annotation in mind, others repurposed to this cause. We hope that these guidelines can offer a starting point for those interested in investigating TE biology.

## Supplementary Information

**Additional file 1.** Tutorial describing, in great detail, each of the steps in the manual curation process of Transposable Elements. Includes instructions for software installation. Suitable for complete beginners.

**Additional file 2.** Videocast showing how to run “make_fasta_from_blast” and how to make multiple sequence alignments.

**Additional file 3.** Videocast showing the process of manual curation of a multiple sequence alignment from a LTR family.

**Additional file 4.** Videocast showing the process of manual curation of a multiple sequence alignment from a LINE family.

**Additional file 5.** Videocast showing how to split a multiple sequence alignment in AliView.

**Additional file 6.** Videocast showing a selection of Unix commands to assist the handling of FASTA flies.

## Data Availability

Data sharing is not applicable to this article as no datasets were generated or analysed during the current study. Code developed for this manuscript is available in the two Github repositories. See Supplementary Methods for more information.

## Abbreviations

EN: Endonuclease
GUI: Graphical user interface
DIRS: *Dictyostelium* Interspersed Repeat Sequence
ICR: Internal complementary region
LINE: Long interspersed element
LTR: Long terminal repeats
MSA: Multiple sequence alignment
PLE: *Penelope*-like element
RM2: Repeatmodeler2
TE: Transposable element
TIR: Terminal inverted repeat
TSD: Target site duplication
RT: Reverse transcriptase
SDR: Split direct repeat
SINE: Short interspersed element

## Glossary

**TE family**: A TE family is a collection of TEs that share a predefined level of homology often described as a percentage of shared sequence identity of all or part of their sequence. In the vast majority of cases, a TE family also represents a group of monophyletic sequences, i.e. members of the same family share a common ancestor. Some exceptions to this exist, such as LTR elements that have high sequence similarity only between their LTR subparts. Wicker et al. [14] suggested the 80–80-80 rule for the incorporation of a TE sequence within a TE family. Briefly, this means that in order to be included in a family, a TE sequence should be at least 80nt long, and share at least 80% sequence identity with the family over at least 80% of their length. TE families can be stored in FASTA files with multiple entries, each corresponding to a member/copy of the family.
**TE consensus**: A consensus sequence is calculated as the most frequent residue at each position derived from a multiple sequence alignment of at least two sequences. These consensus sequences can be calculated for nucleotides or amino acids depending on the nature of the sequences analysed (DNA/RNA or protein). For DNA ambiguous nucleotides (e.g. “R” for “A” or “G”) are sometimes used. The resulting consensus sequence may not exist as such anywhere. In the context of TE biology, each TE family is typically described by a TE consensus (for that family) which is stored as a FASTA sequence. TE consensus sequences are being slowly replaced by probabilistic models (profile-HMMs) that capture the residue variability observed at each position and therefore can store much more information regarding the residue frequencies at each position [34, 71].
**TE library**: This term is used to describe the collection of TE consensus sequences (or TE profile-HMMs) derived from a given analysis done on an organism or group of related organisms. For example, the human TE library comprises all the TE consensus sequences found in the human genome, while the vertebrate TE library contains all TE consensus sequences found in vertebrates. TE libraries are often non-redundant, meaning that one entry represents one TE family, and are stored in FASTA files with multiple entries. A TE library is used to annotate TEs in a genome by sequence homology.
