# Correction: A beginner’s guide to manual curation of transposable elements

**DOI:** 10.1186/s13100-022-00272-4

**Published:** 2022-05-04

**Authors:** Clement Goubert, Rory J. Craig, Agustin F. Bilat, Valentina Peona, Aaron A. Vogan, Anna V. Protasio

**Affiliations:** 1 Canadian Center for Computational Genomics, McGill University, Montreal, Québec Canada; 2 Department of Human Genetics, McGill University, Montreal, Québec Canada; 3 Institute of Evolutionary Biology, University of Edinburgh, Edinburgh, EH9 3FL UK; 4 Departamento de Genética, Facultad de Medicina, Universidad de la República, Montevideo, Uruguay; 5 Department of Organismal Biology, Uppsala University, Norbyvägen 18D, 752 36 Uppsala, Sweden; 6 Department of Pathology, Tennis Court Road, Cambridge, CB1 2PQ UK; 7 Christ’s College, St Andrews Street, Cambridge, CB2 3BU UK

**Correction to: Mobile DNA 13, 7 (2022)**

**https://doi.org/10.1186/s13100-021-00259-7**

Following the publication of the original article [1] the author reported that Additional files 3, 4 and 5 in the published article are corrupted.

The original article [1] has been updated.

## References

[1] Goubert C, Craig RJ, Bilat AF (2022). A beginner’s guide to manual curation of transposable elements. Mobile DNA.

