# Supplementary material for: A beginner’s guide to manual curation of transposable elements
Source: Mob DNA. 2022 Mar 30;13:7. doi: 10.1186/s13100-021-00259-7 (PMC8969392; doi:10.1186/s13100-021-00259-7)
Supplement: Supplementary file 1 — Additional file 1. Tutorial describing, in great detail, each of the steps in the manual curation process of Transposable Elements. Includes instructions for software installation. Suitable for complete beginners. [file 13100_2021_259_MOESM1_ESM.docx]

Supplementary Methods

This document contains supplementary information that accompanies the manuscript entitled: “A beginner’s guide to manual curation of transposable elements” by Clement Goubert, Rory J. Craig, Agustin F. Bilat, Valentina Peona, Aaron A. Vogan, Anna V. Protasio; published in the journal Mobile DNA (add DOI). If you find this material useful, please cite our manuscript.

**Table of contents**

[Software installation.](#_7kv876owt580) 2

[Clone the GitHub repositories TE_ManAnnot and TE-Aid](#_tyv447gx4rhh) 3

[Pfam database](#_3at85h8waqo) 4

[T-coffee installation](#_4ktle1jxypcq) 6

[**Protocols**](#_3q7purpsrie) **6**

[Protocol 0 - Produce a priority table for predicted families.](#_d9uwu8bjxhnv) 7

[Protocol 1 - Extract data from RM2 fasta headers](#_mbyw0we3ygte) 9

[Protocol 2 - Reduce redundancy with `cd-hit`](#_dmtehdk6vm5z) 10

[Protocol 3 - Predict conserved protein domains](#_aatck1c9xsmd) 13

[3a - Using pfam_scan.pl](#_isq4k0hphulg) 13

[3b - Using online servers](#_fs8hagdci36o) 19

[3c - Using `blastx`](#_l7tr8ic3ljwx) 19

[3d - Conserved protein domains found in TEs](#_l8wt23mw1dnf) 20

[Protocol 4 - Obtain the length in nucleotides for each sequence](#_8bpqkdyiy2rv) 21

[Protocol 5 - Run a homology search and count the number of hits with BLAST](#_6bnfalmxxvb) 22

[Protocol 6 - Collect all available data into one table with UNIX commands](#_2cjfh5c786ps) 24

[Protocol 7 - Extract a single fasta sequence from a multi-fasta file](#_nujqq3qae61w) 25

[7A - Extract an individual sequence based on the fasta header.](#_76p26p4xu5oh) 26

[7B - Bulk-extract individual sequences into a directory](#_73wus7b7byp) 27

[Protocol 8 - Find family members in the reference genome](#_jdaaflp5bz8h) 28

[Protocol 9 - Generating and curating multiple sequence alignments (MSA).](#_b8qpvioftdac) 31

[Protocol 10 - Dealing with very large alignments.](#_dz5zq4dgjs74) 33

[Protocol 11 - Families and subfamilies.](#_770w4nvps4uk) 34

[Protocol 12 - Generating consensus sequences and profile-HMM from sequence alignments.](#_2pe7ae3jy7pm) 37

[Protocol 13 - Running TE-Aid](#_23pw5qaejuqt) 40

[Protocol 14 - Visualisation of structural characteristics of TE sequences (dot-plots)](#_3ap2sdb2f8k4) 41

[Protocol 15 - Loops in bash/shell](#_kjknfemt6rzp) 44

[**Appendices**](#_k3ei8vdy10h5) **45**

[I - Penelope-like Elements](#_y3eed92lkish) 45

[II - DIRS elements](#_jq7mmj9sg5ah) 46

[III - Helitrons and Cryptons](#_twfmp5l2a47s) 47

[**Troubleshooting**](#_x2pjt9kwrl8o) **48**

[**References**](#_eeska6p5qldr) **49**

## Software installation.

Basic knowledge of Unix is required for carrying out the protocols described here. This training can be found freely online. We recommend <http://www.ee.surrey.ac.uk/Teaching/Unix/> and <http://swcarpentry.github.io/shell-novice/>.

All of the command line tools can be installed using the package manager Conda. If you are installing it just for the purpose of running software, we strongly recommend that you install the lightweight version of Conda called Miniconda (<https://docs.conda.io/en/latest/miniconda.html>).

Conda/Miniconda allows you to create an environment within your computer and install software that can only be run from within one environment. This is particularly useful when running different versions of software and to avoid incompatibility issues.

To install Miniconda, find the format compatible with your operating system and follow the instructions here:

[https://conda.io/projects/conda/en/latest/user-guide/install/index.html#](https://conda.io/projects/conda/en/latest/user-guide/install/index.html)

Once installed, you can create and install all the necessary programs used in this tutorial using the following command:

| $ conda create -n te_annot -c bioconda cd-hit blast bedtools mafft \ muscle emboss pfam_scan gepard hmmer ucsc-fasplit |
| --- |

*Block 1*

(*) The “\” symbol at the end of each line in a code block indicates that the command continues in the next line. The “\” characters do not need to be typed in the command line.

Once they have been downloaded and installed, you need to load the environment by typing:

| $ conda activate te_annot |
| --- |

*Block 2*

The new command line prompt should have the name of the environment, for example:

| **(te_annot) myuser@mylaptop$** |
| --- |

We recommend that the packages are installed at the same time as the conda environment is created (as written above). It is possible to create the environment first, activate it, and only then install the packages. However, this method may result in inconsistencies given by incompatible python versions.

## Clone the GitHub repositories TE_ManAnnot and TE-Aid

We have developed many scripts for handling and parsing information presented in this tutorial. To have access to these scripts, you will need to clone the GitHub repository.

| $ git clone <https://github.com/annaprotasio/TE_ManAnnot>  $ git clone <https://github.com/clemgoub/TE-Aid> |
| --- |

*Block 3*

Alternatively, you can download the repository directly from GitHub using the same URL above.

In order to run the scripts from TE-Aid, it is necessary to have R [[1]](https://www.zotero.org/google-docs/?F7tNKs) installed in your operating system. This programming language and interface is often installed “from fabric” in many desktop and laptop computers. To test if it is installed, type the following in your terminal window:

| $ which R |
| --- |

*Block 4*

If R is installed, it will return a path, such as: `/usr/local/bin/`. If it returns nothing, it means it is not installed. R is best installed from source, please visit <https://www.r-project.org/> for instructions.

## Pfam database

In order to run `pfam_scan.pl`, you will need to download the profile-HMMs database from their FTP site `ftp://[ftp.ebi.ac.uk/pub/databases/Pfam/](http://ftp.ebi.ac.uk/pub/databases/Pfam/)`. The easiest way to download these files is using the `wget` function that is provided with most operating systems. Follow the instructions below:

1. Create a directory to contain the Pfam database

| $ mkdir Pfam_db  $ cd Pfam_db |
| --- |

*Block 5*

1. Download the database files using `wget` ^^[[1]](#footnote-1)^^

| $ wget ftp://ftp.ebi.ac.uk/pub/databases/Pfam/current_release/Pfam-A.hmm.gz  $ wget ftp://ftp.ebi.ac.uk/pub/databases/Pfam/current_release/Pfam-A.hmm.dat.gz  $ wget ftp://ftp.ebi.ac.uk/pub/databases/Pfam/current_release/relnotes.txt |
| --- |

*Block 6*

Or with `curl`

| $ curl -o Pfam-A.hmm.gz \ ftp://ftp.ebi.ac.uk/pub/databases/Pfam/current_release/Pfam-A.hmm.gz  $ curl -o Pfam-A.hmm.dat.gz \ ftp://ftp.ebi.ac.uk/pub/databases/Pfam/current_release/Pfam-A.hmm.dat.gz  $ curl -o relnotes.txt \ ftp://ftp.ebi.ac.uk/pub/databases/Pfam/current_release/relnotes.txt |
| --- |

*Block 7*

Or alternatively download from the ftp site using an internet browser and move them to the folder you have just created.

1. The zipped files need to be decompressed.

| $ gunzip Pfam-A.hmm.gz  $ gunzip Pfam-A.hmm.dat.gz |
| --- |

*Block 8*

This will make the above files into `Pfam-A.hmm` and `Pfam-A.hmm.dat`.

1. The next step is to “build” the database using the command `hmmpress`. Note that `hmmpress` is part of the `hmmer` package that was installed with conda (above) and is accessible via the `TE_annot` environment. If you have not done so, you need to activate the environment before running `hmmpress` as shown below.

| $ conda activate te_annot |
| --- |

*Block 9*

Then, build the hmm library by running:

| $ hmmpress Pfam-A.hmm |
| --- |

*Block 10*

This command will produce files with extensions `h3i`, `h3m`, `hf3` and `hp3`. The database files and the hmm build files must always be in the same directory in order to operate properly.

**Aliview installation**

To install and run Aliview [[2]](https://www.zotero.org/google-docs/?kiWvQD), follow the instructions in the developers’ website:

<https://ormbunkar.se/aliview/#DOWNLOAD>. An up-to-date Java version is needed to run Aliview; if prompted to do so, please install the latest version of Java that is appropriate for your operating system.

## T-coffee installation

T-coffee [[3]](https://www.zotero.org/google-docs/?y0KaIa) is a powerful tool for multiple sequence alignment generation and manipulation. It has a web interface [[4]](https://www.zotero.org/google-docs/?paqWL9) but many of the more specialised tools, such as gap/inert delietions, are only available through the command line. At the time of writing, the `conda` install package is not available and therefore, `t-coffee` can be installed from source. Visit the `t-coffee` documentation page for instructions on installation for the different systems <https://tcoffee.readthedocs.io/en/latest/tcoffee_installation.html>. If running on an Apple Mac, make sure an update and potentially upgrade of `brew` (a macOSX package installer) is in place. There is a docker image for `t-coffee` here: <https://hub.docker.com/r/cbcrg/tcoffee>

# Protocols

The next section describes the different steps and short programs that are used in the main manuscript. Command line text is entered in console font and shaded in grey. Names of scripts and tools in the body of the text (not the command line) are in `courier font` and variables, such as names of input or output files that are user-defined, will be signposted in *italics*, for example *input.fasta* for in-text referencing or *input.fasta* for command-line instructions.

A set of files including a test chromosome and the RepeatModeler2 (RM2) output from it are included in the Github repository <https://github.com/annaprotasio/TE_ManAnnot> or can be downloaded manually from the repository. These files are used to demonstrate the steps described in this Supplementary Methods document and can be used as a tutorial.

For practical reasons, citations to each of the software/applications used in these protocols are entered the first time the software/applications are mentioned and not subsequently.

Most code blocks in this tutorial take a fraction of a second to run on a standard laptop of desktop computers. The exception is `pfam_scan.pl` which is run as stand-alone and as part of `generate_priority_list_from_RM2.sh`. Consequently, these two commands may take a bit longer to run than the rest, typically in the order of minutes.

## Protocol 0 - Produce a priority table for predicted families.

The generation of a priority table consists of:

- Reducing RM2 output sequence redundancy
- Extracting family classification information
- Calculating sequence length for each predicted family
- Finding and counting protein conserved domains
- Counting number of “credible” blast matches for each family in the genome

There is one protocol (see below) for each of these steps listed above. However, they can all be run by executing the script `generate_priority_list_from_RM2.sh` that comes with the GitHub repo “TE_ManAnnot” (see Software installation) (see inside the `**bin**` folder). This wrapper script will produce the same results as the ones obtained by running protocols 1-6, with the exception that it does not extract data from RM2 fasta headers as it is made by protocol 1.

| $ bash generate_priority_list_from_RM2.sh *rm2-families.fa genome.fa \ path/to/pfam_db_dir path/to/github_repo* |
| --- |

*Block 11*

Note that in order to call the script `generate_priority_list_from_RM2.sh` (this and any of the others from the repository), unless the path has been added to the profile file, the full path to the script needs to be declared. For example, if the repository has been placed in `*~Documents/Github/*`, the call needs to be:

| $ bash \ ~/Documents/Github/TE_ManAnnot/bin/generate_priority_list_from_RM2.sh \ *rm2-families.fa genome.fasta path/to/pfam_db_dir path/to/github_repo* |
| --- |

*Block 12*

See <https://opensource.com/article/17/6/set-path-linux> for more information on how to set up your PATH. The file: *rm2-families.fa* refers to the collection of putative TE families resulting from running RM2, *genome.fa* is the original genome used as input for RM2 or the target genome for which we want to find consensus TEs, *path/to/pfam_db_dir* is the directory where the Pfam database is located and *path/to/github_repo* is the location where the github repo is located. This last variable tells your computer where to find the scripts.

Using our test file examples, this command would be:

| $ bash ~/Documents/Github/TE_ManAnnot/bin/generate_priority_list_from_RM2.sh rm2-families.fa chr.fasta *path/to/pfam_db_dir* ~/Documents/Github/TE_ManAnnot |
| --- |

*Block 13*

The output is a table with the following columns: RM2 fasta header, consensus length (nt), number of good blast hits, number of Pfam conserved domains

Depending on the number of sequences to analyse, this script can take minutes to run whereas most of the subsequent steps are quicker to run.

## Protocol 1 - Extract data from RM2 fasta headers

With the RM2 output, the fasta header of each sequence has information about the TE classification given by the different algorithms used in the TE finding as well as the number of sequences that were used to build the consensus. We can extract this information from the fasta header using the script `rm2_fams2table.pl` (found in the Github repo TE_ManAnnot) on the collections of families present in the RM2 output.

| $ perl rm2_fams2table.pl *rm2-families.fa* |
| --- |

*Block 14*

With our test data:

| $ perl ~/*path_to_repo*/TE_ManAnnot/bin/rm2_fams2table.pl rm2-families.fa |
| --- |

*Block 15*

Remember to change `path_to_repo` with the directory path that leads to where the TE_ManAnnot repository is found **if** the path was not added to your profile beforehand.

This script produces a file with the name `rm2header2table.tab` that looks like:

| ltr-1_family-1 LTR Gypsy Type=LTR 9  ltr-1_family-2 LTR Gypsy Type=INT 4  ltr-1_family-11 LTR Unknown Type=LTR 2  ltr-1_family-12 LTR Unknown Type=LTR 2  rnd-3_family-1 Unknown NA NA 16  rnd-3_family-781 Unknown NA NA 15  rnd-3_family-593 Unknown NA NA 15  rnd-3_family-240 Unknown NA NA 15 |
| --- |

Where the first column is the family name, the second is the Order, the third is the Superfamily (if defined) and the fourth defines for LTR elements whether the “family” is an LTR or INT subpart. The last column represents the number of sequences that were used to build the consensus.

**Note:** One can only use the above Protocol with the RM2 fasta output. Other TE prediction tools will have different formats in their fasta headers.

## Protocol 2 - Reduce redundancy with `cd-hit`

The output of TE *de novo* identification tools is often exhaustive and comprehensive, meaning that in some cases, small variations in sequence can give rise to almost identical or very closely related families of TEs. If guided by the classification suggested by Wicker et al 2007, any two sequences longer than 80nt that share more than 80% identity over 80% of their sequences belong to the same family. This is known as the 80-80-80 rule [[5]](https://www.zotero.org/google-docs/?ZnflYq). This threshold does not represent a biological boundary but gives an empirical cut-off for reproducibility.

Using the RM2 consensus output provided in the test data example, we will attempt to reduce the redundancy of the output by clustering sequences that have more than 80% sequence identity using the software `cd-hit-est` [[6]](https://www.zotero.org/google-docs/?Ndi1CW). Depending on the size of the FASTA file, this process can take some time but it will only have to be run once. Other methods can be used, such as a homology search with Blast [[7]](https://www.zotero.org/google-docs/?CmxL3S), but we find that `cd-hit-est` is an easy and straightforward way of eliminating redundant sequences.

| $ cd-hit-est -i *fasta.in* -o *reduced.fa* -d 0 -aS 0.8 -c 0.8 -G 0 -g 1 \ -b 500 |
| --- |

*Block 16*

The parameters `-i` and `-o` are compulsory (must be declared) as they represent the input file and output file name respectively. The rest of the parameters are optional and the ones shown above have been optimised to reduce library’s redundancy. Briefly:

- “-d” specifies the length of the fasta header (with 0 meaning “keep everything up until the first space)
- “-aS” defines the alignment coverage for the shorter sequence, when set to 0.8 the shorter sequence must have at least 80% of its length matched to the representative sequence, to comply with the 80-80-80 rule
- -c sets the global sequence identity, which is set to 80% to comply with the 80-80-80 rule
- -G defines how identity is calculated, set it to “0” for local sequence alignment and “1” for global alignment. Local (G = 0) ensures that the 80% identity threshold applies only to the aligned stretches between the queried and representative sequences instead of the complete end-to-end (global) alignment of the two.
- -g chooses the clustering algorithm: each sequence is clustered to either first (0) or best (1) representative sequence.
- -b defines the alignment’s bandwidth and has been set to 500. This setting avoids splitting sequences into different clusters where an insertion/deletion causes a break in the synteny of matching identities which, without the indel, is at least 80% of the sequence.

A full list of program options and their descriptions can invoking:

| $ cd-hit-est -help |
| --- |

*Block 17*

Let’s see an example. In our test data (see above), the RM2 output has 404 entries. By running cd-hit-est on this set of sequences, we can cluster a number of them and reduce the number of prospective sequences.

| $ cd-hit-est -i rm2-families.fa -o reduced.fa -d 0 -aS 0.8 -c 0.8 -G 0 \ -g 1 -b 500 |
| --- |

*Block 18*

This process reduces the number from 404 to 227! There is the possibility that we are ignoring some true TE subfamilies in this process, but we can be pretty sure that the ones that are clustering together have enough sequence similarity to each other to be considered part of the same family.

We can inspect the clusters to have a better understanding of which sequences were clustered together. This information is in the file ending with `.clstr`. Cluster 99 shows the following information:

| >Cluster 99  0 77nt, >rnd-1_family-99#Unknown... at 77:1:258:336/-/96.20%  1 106nt, >rnd-1_family-86#Unknown... at 106:1:290:392/-/96.23%  2 159nt, >rnd-1_family-85#Unknown... at 31:159:1:129/+/100.00%  3 52nt, >rnd-1_family-111#Unknown... at 52:1:167:218/-/98.08%  4 608nt, >rnd-1_family-20#Unknown... *  5 143nt, >rnd-1_family-105#Unknown... at 1:143:119:261/+/100.00% |
| --- |

Here, the sequence with name `rnd-1_family-20#Unknown` is the longest and contains in its sequence all the other smaller families that are listed as part of this cluster. Note that they have > 80% nucleotide identity.

If the output from this step is to be taken forwards, we could extract the header information from the `cd-hit-est` output with the script `rm2_fams2table.pl` (see Protocol 1 or below) to produce a new table that matches the sequences we will be working with. Bear this in mind when cross referencing tables and output files.

| $ perl ~/*path_to_repo*/TE_ManAnnot/bin/rm2_fams2table.pl reduced.fa |
| --- |

*Block 19*

## Protocol 3 - Predict conserved protein domains

Many TEs will have open reading frames (ORFs) encoding proteins that are necessary for their transposition. There are many ways in which we can predict conserved protein domains found in TEs, which in turn, can help with their classification into the different superfamilies. Below we present a couple of suggestions. The first recommendation is based on Pfam, a database of manually curated conserved protein domains. This particular step is well integrated with the tutorial that accompanies this supplementary materials. The second recommendation is based on the use of sequence homology search with `blastx`, for which a protein database is required.

### 3a - Using pfam_scan.pl

We can now use the set of new clustered sequences to predict which of them code for a conserved protein domain. We will do this using the Pfam database [[8]](https://www.zotero.org/google-docs/?5DlDO1) but alternative databases can be used for example Interpro [[9]](https://www.zotero.org/google-docs/?G3dMnr) or the Conserved Domain Database [[10]](https://www.zotero.org/google-docs/?lsHyL5).

Most canonical TE superfamilies have a defined set of proteins encoded in their sequence. It is possible to perform a protein domain prediction of any putative TE sequence and to use this feature as a way of prioritising which sequences are most likely to be true TEs. The advantage of this approach is that it can be done automatically using the script `pfam_scan.pl`.

Pfam [[8]](https://www.zotero.org/google-docs/?lBI21e) is a database of manually curated conserved protein domains and `pfam_scan.pl` is a command line tool that allows the user to run the set of profile-HMM available from Pfam against a set of sequences. The output is a table where the first column indicates the TE family and the rest indicates the Pfam domain / profile-HMM matching hit.

Not all TEs have protein conserved domains. Notably, non-autonomous elements can have partial or no conserved domains at all. This should be reason enough not to discard any putative families on the basis of having a zero count of conserved domains.

The first step is to produce a conceptual translation of the nucleotide sequence, that is to say, to obtain the amino acid sequence encoded in the DNA sequence. pfam_scan.pl can translate DNA to peptide as part of the tool but it works more smoothly when we produce the conceptual translations beforehand. We will do this in two ways using two functions of the EMBOSS [[11]](https://www.zotero.org/google-docs/?BpHUqi) package: by obtaining the collection of open reading frames (ORFs) using the function `getorf` and by producing a translation of DNA ignoring any stop codons in between using the function `transeq`. Both approaches have advantages and disadvantages.

To obtain the collection of ORFs,

| $ getorf -sequence *in.fasta* -outseq *out.orf* -minsize 300 |
| --- |

*Block 20*

The option `-minsize 300` will limit the output to those stretches of DNA that run for at least 300 nucleotides long **without** a stop codon. For more information about options in `getorf` type `getorf -help` in your terminal.

Using our test data:

| $ getorf -sequence reduced.fa -outseq reduced.orf -minsize 300 |
| --- |

*Block 21*

This will produce a file called `*reduced.orf*` that contains all the potential ORFs in the nucleotide fasta file. We can now run `pfam_scan.pl` on this file to search for conserved protein domains that are catalogued in the Pfam database. Remember that the `pfam_scan.pl` script requires the location of the Pfam database. For instructions on how to download this database see the section “Software Installation” at the start of this document .

| $ pfam_scan.pl -fasta *out.orf* -dir *pfam_db_dir_location* -outfile \ *out.orf.pf* |
| --- |

*Block 22*

*out.orf*  must be a single or multiple fasta with amino acid sequences.

Using our test example:

| $ pfam_scan.pl -fasta reduced.orf \  -dir *~/Documents/databases/Pfam/Pfam_db* -outfile reduced.pfam |
| --- |

*Block 23*

Note that in this tutorial, the Pfam database files are located in the directory “Documents/databases/Pfam/Pfam_db”. You should change this to reflect the location of the directory where your Pfam database is located.

This step may take some time to run so be patient. The script will produce a file called `*reduced.pfam*`. The first 27 lines are commented and have information about the software and the authors. The lines following the comments are tab-delimited and contain the information regarding the matches to the Pfam HMM database. We can see the first 5 lines of the results with the following command:

| $ cat reduced.pfam \| grep -v "^\#" \| head -5 |
| --- |

*Block 24*

Where `grep -v "^\#" ` excludes all lines that start with “#”. For a more comprehensive explanation of `grep` see **Protocol 7**.

The output from running the above code block should like something like:

| ltr-1_family-1#LTR_Gypsy_2 240 255 239 256 PF00098.24 zf-CCHC Domain 2 17 18 20.7 0.00032 1 CL0511  ltr-1_family-1#LTR_Gypsy_2 493 651 493 651 PF00078.28 RVT_1 Family 1 222 222 90.9 8.2e-26 1 CL0027  ltr-1_family-1#LTR_Gypsy_4 135 248 100 249 PF00078.28 RVT_1 Family 62 206 222 76.6 2e-21 1 CL0027  ltr-1_family-1#LTR_Gypsy_5 72 171 72 171 PF17919.2 RT_RNaseH_2 Domain 1 100 100 110.6 2.9e-32 1 CL0219 |
| --- |

The columns in the output indicate: the query sequence name or id, followed by alignment start and alignment end, envelope start and envelope end. These start and end coordinates are in relation to the query sequence. For example, in the first line, the value for alignment start is 240 and this means that the alignment to the matched HMM (see below) starts at the amino acid number 240 in the query sequence. Envelope and alignment coordinates respond to different probabilities of the HMM match with the sequence (for a full description see “Envelope and alignment domain boundaries” in [[12]](https://www.zotero.org/google-docs/?1CfMm0) or here: <https://pfam-docs.readthedocs.io/en/latest/glossary.html>). The 6th column represents the Pfam accession number for the matched HMM, followed by the HMM name and type (typically Domain or Family). The set of numbers that follow indicate: the start, end and length of the HMM match. If the full-length of the HMM matched the query sequence, then the end and length numbers are the same. The next number is the bit score followed by the e-value. These are two measurements of confidence in the match. The larger the bit score and the smaller the e-value the better the match between the HMM and the query sequence is. The last number is the significance and can be either 0 for non-significant or 1 for significant. When running `pfam_scan.pl` with default parameters this value will always be set to “1”. The last column indicates the Clan to which the profile-HMM belongs. Clans are a Pfam classification that is used to group profile-HMMs that are somehow related to each other, typically because they perform similar functions; for example “RT_RNaseH” (PF17917) and “rve” (PF00665) belong to the clan CL0219 which groups all the Ribonuclease H-like families and domains.

In this example, the first entry family (ltr-1_family-1#LTR_Gypsy) for which at least three ORF have been found (ltr-1_family-1#LTR_Gypsy_2, ltr-1_family-1#LTR_Gypsy_4 and ltr-1_family-1#LTR_Gypsy_5) have matches to the protein domains “zf-CCHC”, twice to “RVT_1”, and to “RT_RNaseH_2”.

We can count how many domains have been predicted for each family using the following “oneliner”.

| $ awk '{if ($6~/^PF/) {print $1}}' reduced.pfam \| sed 's/\#/ /1' \| \  awk '{print $1}' \| sort \| uniq -c \| sort > pf.domains.count |
| --- |

*Block 25*

Where awk '{if ($6~/^PF/) {print $1}}' *can be read as* “for each line, if the 6th column starts with the letters “PF”, then print / return whatever is in the first column of the line”. The output of the first “awk” command is passed onto “sed” (via the “ | “ symbol called “pipe”). Then, sed 's/\#/ /1' *can be read as* “for each line, find the 1st character that matches “#” and replace it with a space”. The output of this query is passed to awk, where by awk '{print $1}' we print the string found in the first column of each line. These are then sorted (with sort ), and counted with uniq -c , then sorted again and printed to a file called “pf.domains.count”. The last 6 lines of the file are shown below.

| 4 rnd-1_family-158  5 ltr-1_family-2  5 ltr-1_family-28  5 ltr-1_family-6  6 ltr-1_family-1  6 ltr-1_family-10 |
| --- |

We eliminated the classification that was added by RM2 in the family name. This is not necessary but will make it easier to collect results into a priority table later on.

To have a complete list with all the families, including the ones without a Pfam match, run the following scripts:

| $ grep '>' reduced.fa \| sed 's/\#/ /1;s/^>//g' \| awk '{print $1}' \  > all.families.names |
| --- |

*Block 26*

In this above block, ` grep “>” reduced.fa ` will take all the lines in the file “reduced.fa” that start with “>” and will pass them on to the next command (via the “pipe” or “ | ” ). The second `sed` command will, for each line, substitute two characters: the first substitution is the first found instance of “#” for a space (indicated as / /) and the second substitution is a “>” symbol - but not any “>” symbol, it will only take a “>” if it is found at the very start of the line (indicated by the metacharacter ^ ) - and will substitute it for nothing (indicated as //). Note two important things in these sed commands, the use of “/1” at the end of the first expression - indicates that only the first occurrence of “#” will be substituted, and the use of “/g” in the second expression - indicates a global substitution. The latter means that the substitution will be applied to every single “^>” character found in the line BUT because there is only one character that can be at the very beginning of the line (that is indicated by ^ ) this “/g” *could* have been replaced by “/1”. The final `awk` command returns only the first column and prints it to a file called “all.families.names”

| $ awk '{if ($6~/^PF/) {print $1}}' reduced.pfam \| sed 's/\#/ /1' \| \  awk '{print $1}' > pf.domains.names  $ cat pf.domains.names all.families.names \| sort \| uniq -c \| \  awk '{print $2,$1-1}' \| sort -k 2 > pf.domains.with0.count |
| --- |

*Block 27*

In the above block, `sort` and `uniq -c` have been explained before (see code blocks 25 and 26). The `awk` command was also briefly explained but in this instance, it includes a mathematical operation. With ` awk '{print $2,$1-1}'` we are asking that for each line, return the second column (indicated by “$2”) followed by the value of the second column minus 1 (indicated by “$1-1”).

Only a fraction of the original clustered sequences have a match against the HMM database. The reasons could be many:

- The putative TE family could be non-autonomous, in which case it could lack protein coding capacity
- The family could code for a protein domain but it is not similar enough to those found in the HMM database and therefore could not be detected
- The family has decayed and lost its coding potential, meaning there are many stop codons in the sequence and a long ORF cannot be found.
- The coding sequence is disrupted by introns and a long ORF cannot be found (not relevant for LINEs and LTR elements).
- The raw RM2 consensus could be an incomplete fragment of the corresponding TE family.

It is possible to repeat this process using the 6-frame translation of the DNA sequence. This can be achieved using the `transeq` function of EMBOSS, which works in a similar way to the `getorf` but with different parameters. The recommended command is:

| $ transeq -sequence *input.fa* -outseq *output.pep* -frame 6 -clean |
| --- |

*Block 28*

The parameter “-clean” makes the stop codon characters appear as “X” instead of “*”. This is particularly useful when using the sequences for downstream analyses where only letters can be read.

Once this is generated, *output.pep* can be searched against Pfam domains. To run pfam_scan.pl on a single sequence, create a file with a single sequence and then run the script with this file as input.

### 3b - Using online servers

Many databases offer web portals that can be used to search the database without the need of a command line or download of the database. The Pfam database itself has a web portal <http://pfam.xfam.org/search/> that takes in amino acid sequences and searches their collection of HMMs. Single sequence as well as batch submissions, of up to 500 sequences (at the time of writing), are possible.

The NCBI Conserved Domains Database [[10]](https://www.zotero.org/google-docs/?Sh2uuw) <https://www.ncbi.nlm.nih.gov/Structure/cdd/wrpsb.cgi> offers more flexibility in that it is possible to submit a DNA sequence instead of amino acid sequences. The maximum number of sequences in a batch submission is 4000.

### 3c - Using `blastx`

An alternative method for identifying conserved domains with sequence homology to known TEs is to perform a homology search with BLAST using our prospective TE sequence as a query against a protein database of known TE peptides. One such database can be downloaded from the RepeatMasker [[13]](https://www.zotero.org/google-docs/?8lgBGn) GitHub repository using `curl`.

| $ curl -o TE_peptide_database.lib \ https://raw.githubusercontent.com/rmhubley/RepeatMasker/master\  /Libraries/RepeatPeps.lib |
| --- |

*Block 29*

(*) The above http address has to be written all in one line.

The next step is to index or “build” the database:

| $ makeblastdb -in TE_peptide_database.lib -dbtype prot |
| --- |

*Block 30*

This will generate a set of files with extensions: “.pin”, “.phr” and “.psq”. The “.lib” file and the index files (“.pin”, “.phr” and “.psq”) should all remain in the same directory. Now, we can use our query sequence to search this database. Since our query sequence is DNA and the database a protein database, we will use BLASTX.

| $ blastx -query *input.fa* -db TE_peptide_database.lib -outfmt 6 |
| --- |

*Block 31*

The `-outfmt 6` option produces a tabular output that is easier to interpret than the default.

### 3d - Conserved protein domains found in TEs

Autonomous TEs encode a minimum set of proteins that are required for their transposition. The list of single domains present in TE sequences is beyond the scope of this work. However, a summary of the most frequently encountered ones is presented. Reverse transcriptases (RT) are the hallmark of Class I retrotransposons and are able to polymerase DNA from an RNA template. The diverse configurations of RT and other domains found in retrotransposons can be indicative of the different diverse superfamilies/families that exist within each order. For example, all autonomous LINEs have RT domains but LINEs of the R2 superfamily have an RT *and* a C-terminal restriction-like endonuclease while LINEs of the RTE superfamily have an RT *and* an N-terminal apurinic-like endonuclease, which is a different type of enzyme. Some superfamilies can have the same combination of domains but in different order. For example, the superfamilies Copia and Gypsy from the LTR order both have the capsid protein GAG and conserved domains corresponding to aspartic protease, integrease, RNAse-H activity and RT activity, but they are encoded in different order. Autonomous DNA Class II transposons with TIRs (terminal inverted repeats) rely on transposases to “jump” from one location in the genome to another. Other transposons classes have more diverse domains (see Appendix in this document). A list of protein domains found in TEs is found in Wicker *et al* 2007 [[5]](https://www.zotero.org/google-docs/?KHCrbo).

## Protocol 4 - Obtain the length in nucleotides for each sequence

Autonomous LTR elements stretch between 3 and >6 kb, while non-autonomous families are about 1-2 kb. LINEs can be more varied but are roughly in the order of 5-8 kb. SINEs are quite small in comparison, up to 1 kb and autonomous TIR (DNA transposons) are mostly ~1-2 kb. Of course, these are just estimates and each organism may have its own unique set of very unusual TEs, such as the case of the giant planaria TEs called Burro, exceeding 25 kb in length [[14]](https://www.zotero.org/google-docs/?s9bLQ6). Adding the length in nucleotides of the prospective family may assist decision making about manual curation.

We provide a script `get_fasta_length.sh` that takes an input fasta file with one or many entries and outputs a space-separated file with two columns: the first column contains the names of the fasta entries and the second column contains the length of each sequence in nucleotides.

| $ bash ~/*path_to_repo*/TE_ManAnnot/bin/get_fasta_length.sh *input.fasta* |
| --- |

*Block 32*

Using our test example, first we keep only the first “word” for each sequence name:

| $ sed 's/\#/ /g' *reduced.fa* \| awk -v OFS='\t' '{print $1}' \  > reduced_short_header.fa |
| --- |

*Block 33*

Then we run that script:

| $ bash ~/*path_to_repo*/TE_ManAnnot/bin/get_fasta_length.sh \ *reduced_short_header.fa* |
| --- |

*Block 34*

## Protocol 5 - Run a homology search and count the number of hits with BLAST

We can obtain the number of significant matches of a given prospective TE family in the genome by running a BLAST homology search and carefully filtering the output to only consider “good” matches.

The first step is to produce the BLAST database of the sequence or set of sequences that will be used as a database. In our case, this database is the genome from which the RM2 families were predicted. We build the database for our test chromosome by running the following command:

| $ makeblastdb -in chr.fasta -dbtype nucl |
| --- |

*Block 35*

By listing the contents of the directory you will now notice that there are some new files with extensions “nin”, “nhr” and “nsq”. These are the accessory files that the BLAST program will need to run the searches. We are now ready to run our homology search for our example files:

| $ blastn -query reduced.fa -db chr.fasta -outfmt 6 \  -out red_vs-genome_blast.o |
| --- |

*Block 36*

This will produce a large number of results with perhaps not very significant hits. We have found it useful to keep blast hits that have at least 50% of the length of the query (i.e. putative TE family) and at least 80% identity. In order to calculate the percentage of the query length that is covered by a given hit, we need to include the query length information in the blast output, which is not part of the default settings (see ref. [[15]](https://www.zotero.org/google-docs/?M1xBno) for more details)

| $ blastn -query reduced.fa -db chr.fasta -outfmt "6 qseqid sseqid \  pident length mismatch gapopen qstart qend sstart send evalue \  bitscore qlen" -out red_vs_genome_blast.o |
| --- |

*Block 37*

This new output will include the query length filed in the last column. We can now filter the results.

| $ awk '{OFS="\t"; if ($3 >= 80 && (($4/$13) > 0.5 )) \  {print $0,$4/$13}}' red_vs_genome_blast.o > red_vs_genome_good_blast.o |
| --- |

*Block 38*

We can now do the same as we did before for the Pfam domains and count how many hits each family has in the genome. Because we cannot guarantee that all families had at least one hit, it is possible that some families are missing from the blast results. With the objective of having a report that also includes 0 counts, we will produce a file that has the first column of blast results, plus we will artificially add one more entry that we can then subtract from the count table:

| $ cat reduced_short_header.fa.len red_vs_genome_good_blast.o \| \  sed 's/\#/ /g' \| awk '{print $1}' \| sort \| uniq -c \| \  awk '{print $2,$1-1}' \| sort > red_vs_genome_good_blast.count |
| --- |

*Block 39*

Note that we only keep the fist part of the family name up to the “#” symbol. This will make it easier to collate all results at the end.

## Protocol 6 - Collect all available data into one table with UNIX commands

So far we have produced data for each prospective family regarding the number of conserved protein domains (as per matched with Pfam), the length of each sequence and the number of “good” blast hits in the genome. We have also produced a table containing extracted information directly from the RM2 output. We can now collate all these output tables into one larger table that we can use to prioritise which families to take forward for manual curation.

The first step is to make sure all files have the same number of lines. We can do this by using the command `wc -l`. If the files we want to collate are named: `reduced.fa.len`, `red_vs_genome_good_blast.count`, `rm2header2table.tab` and `pf.domains.with0.count`, we can run:

| $ wc -l reduced_short_header.fa.len red_vs_genome_good_blast.count \ rm2header2table.tab pf.domains.with0.count |
| --- |

*Block 40*

| 227 reduced_short_header.fa.len  227 red_vs_genome_good_blast.count  227 rm2header2table.tab  227 pf.domains.with0.count  908 total |
| --- |

We first create two tables one with length and blast hits, and the second one with headers and pfam domains counts:

| $ awk 'NR==FNR{a[$1]=$2;next} {print $1,$2, a[$1]}' \ red_vs_genome_good_blast.count reduced_short_header.fa.len \  > cols_len_blast  $ awk 'NR==FNR{a[$1]=$2;next} {print $1,$2,$3,$4, a[$1]}' \ pf.domains.with0.count rm2header2table.tab > cols_head_pf |
| --- |

*Block 41*

Then, the two tables are merged to create the *final_priority.table* file:

| $ awk -v OFS='\t' 'NR==FNR{a[$1]=$2;b[$1]=$3;next} {print \ $1,$2,$3,$4,a[$1],b[$1],$5}' cols_len_blast cols_head_pf > \ final_priority.table |
| --- |

*Block 42*

The resulting table has the following column descriptions:

Family_name

Order

Superfamily

LTR_subpart

Length in nucleotides

No of good blast hits

No of Pfam conserved domains

**Note:** One can only include `red_vs_genome_good_blast.count` if the starting input is from RM2. Other TE prediction tools will have different formats in their fasta headers.

## Protocol 7 - Extract a single fasta sequence from a multi-fasta file

In order to illustrate how to manually curate a given family, the first thing is to isolate the sequence into an individual file. So far, we have worked with the RM2 output or its reduced version (via `cd-hit-est`) and these sequences are found in multi-fasta files. To extract one single fasta sequence from a file, two methods are proposed.

### 7A - Extract an individual sequence based on the fasta header.

Suppose we would like to extract the sequence corresponding to the putative TE family “ltr-1_family-4#LTR”. Since this is the name of the sequence and this string is found in the fasta header, it is possible to use a word matching program to find where this sequence is in the multi-fasta file. To do this, we can use the program `grep` that comes with all Unix distributions. The general syntax is `grep *expression file`* where *expression* is the pattern that we want to find and *file* is the name of the file where we want to find the pattern.

For our example:

| $ grep "ltr-1_family-4#LTR" reduced.fa |
| --- |

*Block 43*

This command will return only the matching line, that is to say, the fasta header of that sequence. If we want to extract the sequence too, we can ask grep to give us the line of the match and one line after the match:

| $ grep -A 1 "ltr-1_family-4#LTR" reduced.fa |
| --- |

*Block 44*

This will return two lines, the header and the next sequence file. If the fasta file is segmented every N number of bases, we will only get the N bases in the first line after the match. To obtain the full length of the sequence, we first need to transform the segmented fasta file to a ‘oneline’ fasta. We can do this using the script `fasta_multi_to_one_line.pl` available in the Github Repository.

| $ perl fasta_multi_to_one_line.pl reduced.fa > reduced.fasta |
| --- |

*Block 45*

This will produce a non-segmented fasta and we can now use `grep -A 1 ` to extract the full length of the sequence”

| $ grep -A 1 "ltr-1_family-4#LTR" reduced.fasta |
| --- |

*Block 46*

### 7B - Bulk-extract individual sequences into a directory

Alternatively, we can extract all sequences individually and save each of them to an individual file. To do this, we will use the tool `faSplit`. This program has many different applications depending on the parameters used. For the purpose of this example, we will use it with the `byname` parameter which will split the multi-fasta file into individual files based on the fasta header (aka name). The syntax is

`faSplit byname *input-multi.fasta destiny_dir/`*

where *input-multi.fasta* is the file containing all the fasta sequences and *destiny_dir/* is a pre-made directory where `faSplit` will write the files, one per sequence.

For our test data, we first need to make a directory:

| $ mkdir ind_seq |
| --- |

*Block 47*

Then we run the script:

| $ faSplit byname reduced.fasta ind_seq/ |
| --- |

*Block 48*

(note that the character `/` **must** be added after the directory name otherwise the individual fasta files will be written to the current directory.)

Running the above gives an error similar to this:

| mustOpen: Can't open ind_seq/ltr-1_family-1#LTR/Gypsy.fa to write: No such file or directory |
| --- |

As it stands, this will give an error because the “/” character is in the fasta header of the `reduced.fasta` file and when it is used as a name in the creation of files, it is understood as a path to a directory. We then need to change the names of the fasta headers so they do not contain this character. For our example, we will exchange the “/” command for a space using `sed` which is installed with all Unix distributions.

| $ sed "s/\// /g" reduced.fasta > reduced_namechg.fasta |
| --- |

*Block 49*

And now:

| $ faSplit byname reduced_namechg.fasta ind_seq/ |
| --- |

*Block 50*

Watch this **Supplementary Video 1** for a screencast demonstrating the steps shown in this Protocol.

## Protocol 8 - Find family members in the reference genome

In order to build/rebuild a consensus sequence, it is necessary to find the members of the putative TE family in the reference genome. To this end, the sequence representing the TE family is used as a query in a `blast` search against the reference genome. Because the putative TE family could be fragmented, the boundaries of the blast hit are extended/moved up- and down-stream of the blast hit coordinates. This step aims at capturing as much of a full length of the TE as possible. The last step is to extract the nucleotide sequence from these locations in the reference genome and write them to a file for later manipulation. These three steps are wrapped up in the script `make_fasta_from_blast.sh` which is available from the GitHub repository. In order to be able to run this script, the following software needs to be installed and accessible:

- Blast
- Bedtools
- Samtools

For instructions on how to install software, please refer to the “Software Installation” section found at the start of this document. We strongly recommend that a dedicated Conda environment is set up.

`make_fasta_from_blast.sh` uses third party software, which come with their own requirements. To run `blast`, a blast database needs to be created and similarly, the two `bedtools` functions called from the script require an auxiliary file that contains the reference’s chromosome names and lengths. `make_fasta_from_blast.sh` will create these auxiliary files automatically and, as long as they are not deleted or moved to another directory, they will not be generated again. The output of `make_fasta_from_blast.sh` is a multi-fasta file with all the sequences of the best blast hits and their flanking extensions.

The syntax for `make_fasta_from_blast.sh` is:

`make_fasta_from_blast.sh <genome> <fasta.in> <min_length> <flank>`

An example run could be:

| $ bash make_fasta_from_blast.sh *genome.fa putative-TE-1.fa 400 1000* |
| --- |

*Block 51*

Note that in the above example:

- *genome.fa* is the reference genome,
- *putative-TE-1.fa* is a single fasta sequence for example, one of the families that form the output of RM2,
- *400* is the minimum length of the blast hit which means that any hits below this length will not be considered, and
- *1000* is the number of bases that the blast hit will be extended up and downstream.

If the blast database field is not present, the script will attempt to generate it and will show the following text: “Blast database doesn't exist. Running makeblastdb, this can take some time”. The same for the genome.length file. If it does not exist, it will try to generate it and will display the message: “File with genome lengths not found. Making it now, this can take some time”

An important aspect of running `make_fasta_from_blast.pl` is to filter hits by size. This filter can be applied with the third “flag” or <min_length>. In the example above, <min_length> was set to “400” (see above). When this value is set to “0”, the script automatically sets the threshold to half the length of the query. So, if the query is 1000 bp long, by setting <min_length> to 0 all subject hits returned as part of the multi-fasta will be at least 500 bp long, as shown in the example below.

To run `make_fasta_from_blast.sh` with one of the putative families from our test example:

| $ bash make_fasta_from_blast.sh \  chr.fasta ind_seq/ltr-1_family-4#LTR.fa 0 500 |
| --- |

*Block 52*

Note that `ind_seq/ltr-1_family-4#LTR.fa` was created using the instructions in **Protocol 7**.

This will produce a file called `ltr-1_family-4#LTR.fa.blast.bed.fa` that has all the hits in the genome that meet the requirements specified in the blast call as “good hits” (with e-value cut off 1e-20).

An additional script `make_align_from_blast.sh` provided with the GitHub repo includes an alignment step (`mafft`) after retrieving sequences from the genome, saving time.

See **Video 1** for a screencast on the use of the scripts explained in this Protocol.

## Protocol 9 - Generating and curating multiple sequence alignments (MSA).

The next step is to align the sequences of the prospective members of the TE family. An MSA is required for the generation of a consensus sequence (see **Protocol 11**). There are a number of different aligners that can be used. We will use MAFFT [[16]](https://www.zotero.org/google-docs/?PQYdw2) in our example. To run `mafft` with default parameters:

| $ mafft *input.fa* > *output.maf* |
| --- |

*Block 53*

Continuing with our test example:

| $ mafft ltr-1_family-4#LTR.fa.blast.bed.fa > ltr-fam4.maf |
| --- |

*Block 54*

Other fast sequence aligners are MUSCLE [[17]](https://www.zotero.org/google-docs/?Sjf6YF), Clustal [[18]](https://www.zotero.org/google-docs/?9I1aZA) and many more. Generating an accurate MSA is critical for the generation of high-quality TE families and libraries. A recent benchmark study [[19]](https://www.zotero.org/google-docs/?ZG5Wvt) clearly indicates that not all aligners perform equally, especially when low and medium divergent sequences are included, as is the case in the production of TE families. During the manual curation process we may be required to run sequence alignments many times and sometimes iteratively. For this reason, we will prefer fast tools even at the cost of accuracy.

Finally, the alignment can be visualised in Aliview or alternative software and manually curated. For more details on these processes please watch **Video 2** and **Video 3**.

One very laborious process is the removal of gaps from the alignment. Most outputs from running the MSA steps will produce alignments that have rare insertions. These can vary from single nucleotide insertions (perhaps found in 1 or 2 sequences in the alignment) to large insertions of 500 nt or 1 kb long. Very large insertions can sometimes include nested TEs and although these are not rare, they are not informative in the generation of a consensus sequence.

It is possible to remove gaps manually using Aliview, by selecting one or many consecutive columns that are deemed non informative and deleting them from the alignment. This is an extremely labour intensive task that can sometimes lead to deleting columns that are not intended for deletion. Alternatively, a consensus making tool that can process gaps could be used (i.e. a gap can be the consensus “base” and can easily be removed later on the command line or in a text editor), such as the online Advanced Consensus Maker (https://www.hiv.lanl.gov/content/sequence/CONSENSUS/AdvCon.html). The drawback of the said tools is that, at the time of writing, it is only accessible from a web-portal. A more straightforward, automatic and reproducible way of removing insertions or gaps is by applying the “rm_gap” function from the T-COFFEE [[3]](https://www.zotero.org/google-docs/?QGcqYJ) package. `t-coffee` is a very versatile collection of algorithms that focuses on solving various problems arising from multiple sequence alignments and their analyses. It is possible to use `t-coffee` as an aligner *per se* and instead of `mafft or `muscle` (as long as there is consistency throughout the analyses) but in this instance, the use of `t-coffee` is illustrated for the purpose of removing non-informative rare insertions or gaps.

| $ t_coffee -other_pg seq_reformat -in *fasta.aln* -action +rm_gap 80 |
| --- |

*Block 55*

In this case, `*fasta.aln*` is a previously aligned multi-fasta file as the one obtained from running `mafft`. All other arguments in the command are options that are passed on to `t-coffee`. Of note, “ +rm_gap 80 ” removes residues occurring in a column that contains 80% or more gaps (for a complete description of all T-COFFEE arguments and options please see <https://tcoffee.readthedocs.io/en/latest/index.html>). Although reproducible, it is important to note that automating this process can sometime have unintended consequences (e.g. an alignment may feature a lot of gaps if many much shorter nonautonomous copies of a family were retrieved together with fewer copies of the target autonomous family due to homology at the termini), and we would always recommend quickly comparing the unprocessed and processed alignments to make sure that the removal of gaps was desirable.

The curated alignments can be used for a number of downstream analyses. For example, they can be used to generate hidden markov models (HMM) for submission to the public repository Dfam [[20]](https://www.zotero.org/google-docs/?KHaVUA) or to find subfamilies with the TE families. Regardless of the ultimate use of the alignment file, it is important to **save the alignment file** in a safe and backed up location.

## Protocol 10 - Dealing with very large alignments.

Because the final set of sequences retrieved using blast hits could be relatively long, the speed of the alignment process might be compromised, particularly for putative families with hundreds or even thousands of instances in the genome. Thus, if a relatively large number of families in the sample behave this way, the alignment could be a time consuming step even with fast alignment tools, especially if no access to a computer cluster is available.

To speed up this process and to obtain a representative set of sequences from these families, a subset (of for example 100 to 200 sequences) could be randomly selected for alignment from those large multi-fasta files. However, it should be taken into consideration that the transposon family’s genomic instances usually include different structural variants or truncated elements which may vary significantly in size [[21], [22]](https://www.zotero.org/google-docs/?OPPuxx). As a consequence, it might be preferable to get the largest sequences for the alignment so that full-length and truncated copies could be entirely recovered from the genome by downstream analysis with the consensus sequence obtained from that alignment. A possible strategy to address the previous considerations is followed by the provided script `readyForMSA` whose syntax is: `readyForMSA <file.bed.fa> <max> <keep_largest>` where *file.bed.fa* corresponds to a multi-fasta file from the output of the script `make_fasta_from_blast.sh` **(Protocol 8)**, *max* is a positive integer set by the user corresponding to the maximum number of sequences to be aligned and *keep_largest* is also a positive integer, ranging from 0 to *max*, which allows the user to keep the largest sequences from the original input.

In the following example, if *input.bed.fa* is a file which contains more than 100 sequences, then an output called `input.bed.fa.rdmSubset.fa` is obtained:

| $ bash ready_for_MSA.sh *input.bed.fa* *100 25* |
| --- |

*Block 56*

This output is a fasta file including the 25 largest sequences plus another 75 (100-25) that are randomly selected from the remaining sequences in the *input.bed.fa* file. This bias towards the largest sequences could be prefered by the user, particularly when dealing with families with a high proportion of truncated genomic instances. However, a possible drawback for this strategy is that it might generate a bias towards genomic instances containing large insertions such as nested TEs. Whereas rare insertions are normally eliminated when curating the alignment, this bias increases the chances of generating a consensus including these insertions, depending on the characteristics of the particular family as well as the value assigned to the *keep_largest* parameter. Taking these considerations into account, the user might prefer to not keep the largest sequences. This could be done just by setting the *keep_largest* parameter to ‘0’, so that all the 100 sequences, in the above example, are randomly selected from the input file. Finally, in addition to the fasta file, a file named `input.bed.fa.rdmSubset.len` is created, which contains the headers and lengths of the corresponding subset of selected sequences.

## Protocol 11 - Families and subfamilies.

After the manual curation of the alignments (following **Protocol 9**), it is advisable to cluster the obtained consensus sequences by similarity at the nucleotide level to divide them into categories of family and subfamily and, in this way, reducing potential redundancy within the TE library. For family level curation of libraries, as it has been described in this tutorial, we recommended using `cd-hit-est` with `-c 0.8` and `-aS 0.8`, which are stringent parameters and will cluster highly similar TE consensus sequences into families. If more diversity and thinner classification is desired, it is possible to identify subfamilies within families.

To find subfamilies within families, it is necessary to go back to the alignment file. For family level curation of libraries, as it has been described in this tutorial, we recommended using `cd-hit-est` with `-c 0.8` and `-aS 0.8`, which are stringent parameters and will cluster highly similar TE consensus sequences into families. This level of detail is enough for most applications. However, if more diversity and thinner classification is desired, it is possible to identify subfamilies within families.

Families and subfamilies classification of TEs can be mirrored to genera and species classification of organisms and it is possible to distinguish these two categories by performing an “*all vs. all*” alignment of the consensus sequences and by filtering alignments following the 80-80-80 rule [[5]](https://www.zotero.org/google-docs/?65ulht) for families and the 80-95-98 rule for subfamilies [[23]](https://www.zotero.org/google-docs/?wU9xS4). Other cut-offs have been proposed such as 90/90 [[5]](https://www.zotero.org/google-docs/?A0iyAL). Briefly, family members are at least 80 nt long, share similarity of at least 80% of residues over at least 80% of their length (80-80-80 rule). Moreover, two sequences are part of the same subfamily if 98% of their length exceeds a level of similarity of 95% (80-95-98 rule). Identification of consensus sequences belonging to the same subfamily allows to keep in the library only one consensus sequence per subfamily, reducing the TE library redundancy.

To obtain the full diversity of subfamilies from the curated families, the curated alignments need to be reprocessed using slightly different clustering parameters. We previously used `cd-hit-est` to cluster sequences with at least 80% identity over 80% of the length of the shortest sequence using the following command.

| $ cd-hit-est -i *input.fa* -o *reduced.fa* -d 0 -aS 0.8 -c 0.8 -G 0 -g 1 \  -b 500 |
| --- |

*Block 57*

This was applied to the whole library and on occasions, the combined “curated” and “raw” libraries. To identify subfamilies within the library we recommend using slightly different cd-hit-est parameters that correspond to the 80-95-98 rule but using the alignments as input.

| $ cd-hit-est -i *fam1_alignment.fa* -o *fam1_subfams.fa* -aS 0.98 -c 0.95 \  -G 0 -g 1 |
| --- |

*Block 58*

To illustrate this application, we selected three sequences from rm2-families.fa and placed them in a directory within the tutorial_data folder in the Github repository TE_ManAnnot. The file sequences.fasta contains three entries that are quite similar to each other. This group of sequences form one family but two subfamilies. Running:

| $ cd-hit-est -i sequences.fasta -o families -d 0 -aS 0.8 -c 0.8 -G 0 \  -g 1 -b 500  $ cat families.clstr |
| --- |

*Block 59*

produces one cluster:

| >Cluster 0  0 757nt, >seq1... at 1:757:153:833/+/87.34%  1 363nt, >seq2... at 1:363:12:374/+/99.45%  2 914nt, >seq3... * |
| --- |

While running:

| $ cd-hit-est -i sequences.fasta -o subfams -d 0 -aS 0.98 -c 0.95 -G 0 \  -g 1  $ cat subfams.clstr |
| --- |

*Block 60*

produces two clusters:

| >Cluster 0  0 363nt, >seq2... at 1:363:12:374/+/99.45%  1 914nt, >seq3... *  >Cluster 1  0 757nt, >seq1... * |
| --- |

`seq1` has 87% residue identity with respect to `seq3` and therefore it is included in the family but excluded from the subfamily categorisation. It is informative to retain the family name when naming subfamilies, for example by adding a letter to distinguish between subfamilies (Family *fam1_species* could have two subfamilies named *fam1a_species* and *fam1b_species*).

## Protocol 12 - Generating consensus sequences and profile-HMM from sequence alignments.

Once the sequence alignment has been curated (see main text and screencasts) and saved (**please do not forget to save your modified alignment**), a consensus sequence can be derived from the MSA. There are many programs that can generate a consensus from a MSA. For our example we will use the program `cons` that is included in the EMBOSS package.

| $ cons -sequence *input.aln* -outseq *output.cons* |
| --- |

*Block 61*

`-sequence` and `-outseq` are compulsory parameters that need to be defined. There are other options that can also be specified and will affect the way `cons` decides how to call the consensus base at each column of the alignment. In particular, the `-plurality` option can be changed to a lower value (for example, 0.1 - 0.3) resulting in less ambiguous positions (less Ns). The best way to understand this function is to produce a test set of sequences and run the same program with different options and check the output. For assistance with this task, we have created a short artificial MSA and ran `cons` with different parameters.

|  | 1 | 2 | 3 | 4 | 5 | 6 | `cons` command |
| --- | --- | --- | --- | --- | --- | --- | --- |
| >seq1 | A | A | G | A | A | - |  |
| >seq2 | A | G | A | T | A | A |  |
| >seq3 | - | A | G | T | - | - |  |
| >seq4 | - | G | A | T | T | - |  |
|  |  |  |  |  |  |  |  |
| default | a | a | g | T | a | n | cons -sequence *input* -outseq *output* |
| identity=1 | a | a | g | T | a | n | cons -sequence *input* -outseq *output* -identity 1 |
| identity=2 | a | a | g | T | a | N | cons -sequence *input* -outseq *output* -identity 2 |
| identity=3 | N | N | N | T | N | N | cons -sequence *input* -outseq *output* -identity 3 |
| identity=4 | N | N | N | N | N | N | cons -sequence *input* -outseq *output* -identity 4 |
| plurality=1 | a | a | g | T | a | a | cons -sequence *input* -outseq *output* -plurality 1 |
| plurality=2 | a | a | g | T | a | n | cons -sequence *input* -outseq *output* -plurality 2 |
| plurality=3 | n | n | n | T | n | n | cons -sequence *input* -outseq *output* -plurality 3 |

In this table, rows indicated >seq1 … >seq4 represent the four sequences that form the alignment, and the columns 1-6 show the base for each sequence at each given position in the alignment. The rest of the rows (default, identity=1, etc) show the results obtained when running `cons` with the indicated parameters as shown on the right hand side columns. For example, the row named “default” shows results of running `cons` with default parameters, i.e specifying only `-sequence` and `-outseq` while “identity=1” shows the result of running `cons` with default parameters **except** adding `-identity 1`. In summary, if the objective is to capture all insertions without registering “n” for the same, `-plurality` should be set to 1. This parameter tells `cons` to accept a minimum of 1 base per column and give the consensus this base even if it is the only base in the column. If, on the contrary, the objective is not to record insertions, `cons` can be run with default parameters producing an “n” in those positions where there is a rare insertion (position 6) but the identity of the same cannot be assigned with certainty. It is possible to remove the “n” characters with `tr`, but this action could introduce frameshifts in the open reading frame.

| $ cat *output* \| tr -d `n` |
| --- |

*Block 62*

Notice that given an equal number of the same bases in a given position, as it happens for positions 2 and 3 where the frequency of bases is the same but in different order, `cons` defaults to assign the base that appears in the first sequence. Alternatively, the EMBOSS tool `consambig` could be used, which would result in the ambiguous nucleotide “R” (meaning “G or A”) being incorporated at positions 2 and 3. More fine-tuned parameterisation can also be achieved with Advanced Consensus Maker, although this is only available as a web-based tool and is not covered in detail here. The EMBOSS `cons` function also has a web server tool that can be found here: <https://www.ebi.ac.uk/Tools/msa/emboss_cons/>

After generating a consensus sequence, it is possible to find open reading frames, generate the conceptual translation (amino acid sequence from DNA) of the consensus and predict conserved protein domains. These steps are described in **Protocol 3**.

One disadvantage of consensus sequences is that they are limited in their capacity to retain information about the variations occurring in each position. To overcome this issue, it is possible to generate profile hidden markov models or **p-HMMs**. These probabilistic models can be used to search databases and to also derive a consensus sequence. They contain more information in them than the consensus sequence.

Once a MSA has been generated, producing a p-HMM is straightforward using the program `hmmbuild`, as shown in the example below:

| $ hmmbuild *hmmout.stk input.aln* |
| --- |

*Block 63*

Where input.aln is the MSA and hmmout.stk is the p-HMM output file (stk stands for “stockholm” which is the file format used for annotating p-HMMs. The output is a flat file (i.e. human-readable) matrix type file. HMMs can be used as inputs to search a fasta database using the tool `hmmsearch` (included in the conda environment te_annot) and can also be used to submit TE families to the database Dfam. [[20]](https://www.zotero.org/google-docs/?MYRy5U)

## Protocol 13 - Running TE-Aid

TE-Aid is a unix/R program aimed to provide useful information about a given TE consensus. TE-Aid requires as input a consensus sequence (typically from the TE library under curation) and the reference genome. The program requires Blast+, R and EMBOSS getorf to be accessible in the user path.

TE-Aid produces 4 figures and optional tables (**Figure 5**). The consensus genome is searched against the reference genome with blastn. Hits are reported on the top left as horizontal segments along the consensus sequence (x axis); the divergence between a given genomic hit and the consensus is reported on the y axis. The hits are piled-up and a second graph in the top right is made, representing the genomic coverage (represented by the multiple genomic hits) along the consensus sequence. A self dot-plot is produced by searching the consensus sequence against itself with blastn (bottom left). This step allows the identification of specific features, such as TIRs, LTRs, or tandem repeats for example, the coordinates of which can be extracted from the optional output tables. Finally, potential ORFs are extracted in the 6 frames with EMBOSS getorf, and the resulting predicted peptides searched with blastp against a curated library of TE proteins (<https://raw.githubusercontent.com/rmhubley/RepeatMasker/master/Libraries/RepeatPeps.lib>). TE peptide hits and possible TIRs or LTRs are printed on the final figure (bottom right).

A full description, the installation guidelines and a tutorial for TE-Aid are provided in the GitHub repository (​<https://github.com/clemgoub/TE-Aid>). Once installed a given TE consensus *TE_consensus.fasta* can be searched with the following command:

| $ ./TE-Aid -q *TE_consensus.fasta* -g *genome.fast*a -o *outputfolder* |
| --- |

*Block 64*

Here, -o will create a folder “outputfolder” containing the results. To add the optional tables for further analysis, the options -t (self dot-plot, protein hits) or -T (sample + genomic hits [can be large]) can be set. This is useful to further refine the results, estimate copy numbers, or get the precise coordinates of features such as TIRs and LTRs.

See Supplementary Text file for a comprehensive gallery of typical TE-Aid outputs.

## Protocol 14 - Visualisation of structural characteristics of TE sequences (dot-plots)

In this section we cover two tools for displaying dot plots of sequences. Both are based on the self-alignment of the TE sequence.

In brief, a dot-plot tool produces a matrix with the sequence A on the columns and sequence B in the rows like the figure below. It then compares all-to-all positions and marks those instances where the row and column entry are the same. This leads to a matrix or 0 and 1.

|  | A | C | T | G |
| --- | --- | --- | --- | --- |
| A | 1 | 0 | 0 | 0 |
| C | 0 | 1 | 0 | 0 |
| G | 0 | 0 | 0 | 1 |
| T | 0 | 0 | 1 | 0 |

When the sequence A is identical to sequence B, there is a diagonal of 1s. This is going to be the case every time a “dot plot to self” is performed but it will also reveal other structural characteristics for example, if the same “word” is found at the start and at the end of the sequence, this will produce an image like the dot-plot below, which was generated using Gepard and an LTR retrotransposon. Notice how the LTR subparts are visualised in the top right and bottom left corners. This sequence is the same at the start and at the end of the consensus sequence, therefore they form this pattern.

Gepard can be operated from a GUI and the input files can be selected from drop down menus. The plot it generates is dynamic i.e. it assists zoom in/out functions as well as printing a static image to a file.


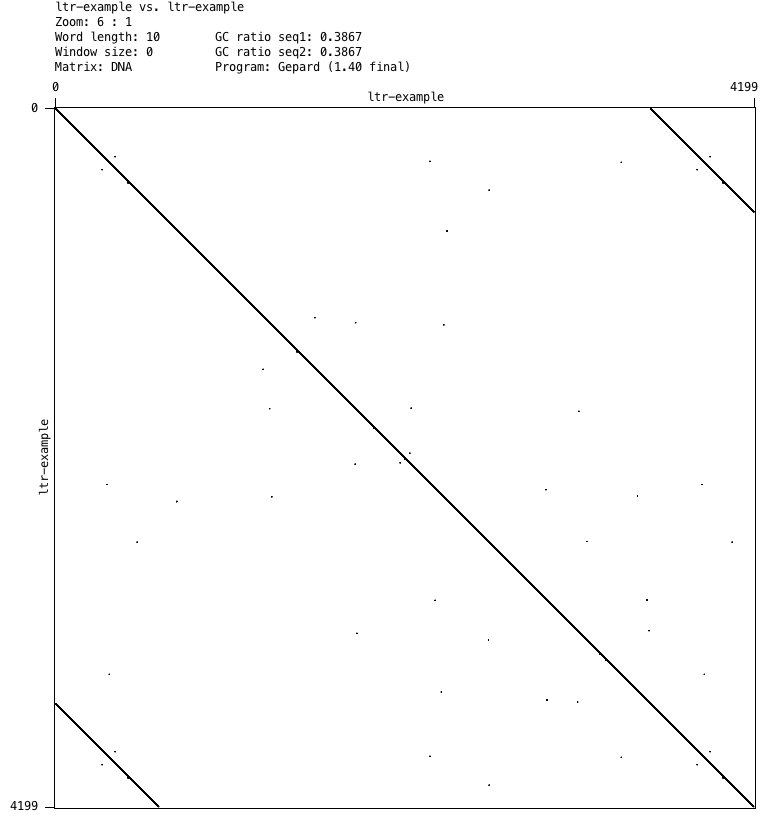


Figure - Classical dot-plot from a self-alignment of a LTR element.

For practical reasons, dot-plot algorithms will show a match (following our example from the table above that would be equivalent to recording a 1) only when a set of 10 or more letters have a match in the comparing sequence. This is called the “word length” and a match of 10 consecutive letters (i.e. a word) is less likely than a 1 to 1 match. It is not practical to compare all individual positions and it is quite computationally intensive. In Gepard, it is possible to change the word length from its default setting of 10 (accessible via Advanced Mode / Plot). A shorter word length will produce more matches and a noisier plot but will be able to identify shorter sequences that are found multiple times in the fasta entry, for example, terminal inverted repeats that are often less than 10 nucleotides long. However, reducing the word length will take more computer resources and will take longer to run, although this is perhaps not a problem for fasta entries of the order of 5-8 kb long.

An alternative online tool is “Align two sequences” from the NCBI Blast server. At the time of writing, this tool can be accessed via the web page <https://blast.ncbi.nlm.nih.gov/Blast.cgi>, then selecting “Nucleotide Blast” and selecting the checkbox “Align two or more sequences”. For an alignment to self, the same sequence must be entered in the top and bottom boxes. The default aligner in this web application is megablast and we recommend that this is changed to blastn. The output in the dot-plot tab should look similar to the one above. Dot plots can also be output by the online MAFFT server (<https://mafft.cbrc.jp/alignment/server/>).

## Protocol 15 - Loops in bash/shell

All the command-line tools and scripts that we have seen in this tutorial can be run in “loops” where the same script or action is applied to all the files in a directory with a shared characteristic. Let’s imagine we want to apply the tool `transeq` to all 30 individual DNA sequences in a directory. I have two options: a) to run 30 commands calling `transeq`, one per DNA file, or b) to run one command that loops on all the 30 files in the directory.

The basic loop call is:

| $ for i in *list.of.files*; do *something*; done |
| --- |

*Block 65*

If the DNA files are called DNA1.fa, DNA2.fa …. DNA30.fa, and the objective is to translate them using `transeq`, the command could look like this:

| $ for i in DNA*.fa; do transeq -sequence $i -outseq $i.aa; done |
| --- |

*Block 66*

In this loop, DNA*.fa stands for all the possible matched to that name, with `*` matching anything any number of times. The first `i` is the variable, that for each loop, it will acquire the value of the name of one file. In Unix, variables are assigned their value without any symbol (i.e, just ` i `) but when the variable is called, we use `$i`. This is why in the “do” block, it is called $i and is used as the input for `transeq`. Note that we are also using the variable to define the `-outseq` value, in this case the file name for the output. We ask the code to call it the same as the input, that is `$i` but with the added extension `.aa`.

To practice this new skill, you can use individual 5-6 sequences from the `rm2_famlies.fa` file (see **Protocol 7** for how to extract individual sequences) and use a loop command to translate them into amino acids. Try then to use `getorf` instead of `transeq`. What other actions could you streamline with this new tool? Happy coding :-)

# Appendices

## I - *Penelope*-like Elements

PLEs represent their own unique order of retrotransposons, distinct from LINEs, LTRs and DIRS. Although relatively poorly studied, recognition of the phylogenetic and taxonomic diversity of PLEs is increasing as more diverse eukaryotic lineages are targeted for genome sequencing. Autonomous PLEs are characterized by the presence of an ORF encoding reverse transcriptase (RT) and a GIY-YIG endonuclease (EN), which can be located at the N or C terminus [[21], [24]](https://www.zotero.org/google-docs/?74ZG7q). The GIY-YIG EN is diagnostic of PLEs, although PLEs lacking EN also exist, which are generally limited to telomeric insertions [[25]](https://www.zotero.org/google-docs/?Im3V6g). Additionally, the RT of PLEs is sufficiently distinct from LINEs and LTRs to be diagnostic in database queries. Most PLE insertions are 5’ truncated, and upon first viewing an alignment of PLE copies it may resemble that of a LINE. However, PLEs exhibit several specific peculiarities. Full-length insertions are characterised by a structure called a pseudo LTR (pLTR), which is formed by the insertion of a second PLE copy in tandem, which itself is generally 5’ truncated. When viewed as a self dotplot, this combination of a full-length copy plus a partial tandem copy resembles an LTR (See Supplementary Text, TEAid gallery). The second copy is rarely full-length, but when it is, it can even be followed by a third copy. To further complicate matters, upstream of the pLTR an additional inverted and 5’ truncated copy can occasionally be observed, which then resembles a TIR. Due to the complex nature of tandem insertion and the frequent 5’ truncation of copies, PLEs derived from automated predictors are regularly truncated prior to the pLTR. Indeed, although tandem insertion and the pLTR is a functional requirement of activity , it is recommended to produce a consensus sequence of only the main body of a single insertion. Certain PLE superfamilies are associated with microsatellite insertion targets, and the precise termini of copies can sometimes be difficult to determine due to the presence of a “tail” – a 30-40 bp extension found at either end of the insertion. Finally, unlike other retrotransposons PLEs can contain introns, although in most families these do not disrupt the main ORF encoding RT-EN. Nonautonomous PLEs have been observed, which can form multiple head-to-tail tandem insertions [[24]](https://www.zotero.org/google-docs/?hxhNtU), and SINE-like retrozymes that likely utilize PLE proteins for their transposition have also recently been described in animals [[26]](https://www.zotero.org/google-docs/?XU0OAL).

## II - DIRS elements

DIRS are another distinct group of retrotransposons, which can be identified by their unique termini and lack of TSDs. The first of these elements, described in the slime mould *Dictyostelium discoideum*, was named *DIRS-1* [[27]](https://www.zotero.org/google-docs/?0rjLTG) and since then related TEs have been referred to as “DIRS” inferring the meaning “DIRS-1-like element”. As introduced in the main text, two distinct types of DIRS structural organisations exist. The first type (Order: DIRS, Superfamily *DIRS-like* [[5], [28]](https://www.zotero.org/google-docs/?IR0N3r)) has inverted terminal repeats (ITRs), which can be imperfect, and a short internal complementary region (ICR) located at the 3’ of the internal region (i.e. between the final ORF and 3’ ITR). The 5’ of the ICR is complementary to the beginning of the 5’ ITR, while the 3’ is complementary to the end of the 3’ ITR. The second type, which can be further split to three putative superfamilies, is characterised by split direct repeats (SDRs), where there are two pairs of direct terminal repeats termed A1 and A2, and B1 and B2. The most common structural representation is “A1-coding sequence-B1-A2-B2”, although other configurations exist. Examples of both types are represented in the TE-Aid gallery. The RT domain of DIRS is phylogenetically related to that of LTR elements, however DIRS lack the integrase (INT) and proteinase (PROT) domains and instead contain a tyrosine recombinase (YR) domain. The basic organisation of autonomous DIRS features three ORFs, which often overlap and encode a GAG-like domain, the RT/RNAseH domain, and the YR domain, respectively. Additional domains and ORFs can be present, for example a methyltransferase domain as part of the RT/RNAseH ORF is a conserved feature of certain superfamilies.

## III - Helitrons and Cryptons

Cryptons and Helitrons are DNA elements with small or no TIRs, often no TSDs, and low copy numbers, so determining termini can be very difficult. They are generally only defined by a protein encoding a replication initiator-like (REP) domain and helicase (Helitrons) or YR domain (Cryptons). As with the LINEs, it is recommended to use a partial sequence that covers these domains unless many copies exist and the termini can be confidently defined. Both Helitrons and Cryptons may feature introns, further complicating their annotation. The TE *de novo* pipeline EDTA [[29]](https://www.zotero.org/google-docs/?UeQ6Bg) incorporates *HelitronScanner* [[30]](https://www.zotero.org/google-docs/?fjUZPM), a Helitron search algorithm, which is based primarily on the structural characteristics of canonical Helitrons (i.e. the more recently described *Helitron2* superfamily [[31]](https://www.zotero.org/google-docs/?W5KLO8) may not be detected using this approach). Combining *HelitronScanner* and the existence of a REP and/or helicase domain would be a good approach to reduce the number of hits that will require manual curation. In combination with the terminal structural motifs present among different Helitrons (see main text), there is substantial variation in total coding capacity and many Helitrons feature additional domains or entire auxiliary genes [[32]](https://www.zotero.org/google-docs/?0qBfwx). Similarly, the presence of a YR domain without any of the other domains (RT, RNAseH, etc.) or structural motifs associated with DIRS (see above) would be a good indication of a potential Crypton. Cryptons can also feature additional domains and genes, some of which may be informative of superfamily-level classification [[33]](https://www.zotero.org/google-docs/?6Shh25). The terminal motifs of Helitrons and Cryptons are detailed in the main text.

# Troubleshooting

Problem:

- My computer cannot find the command, gives an error that says something like: “-bash: generate_priority_list_from_RM2.sh: command not found”

Solution:

- Provide the full path to the location of the script, for example “~/Desktop/TE_ManAnnot/bin/generate_priority_list_from_RM2.sh” followed by the argument for the script. For quicker use, you can set the path of the scripts’ location. See <https://opensource.com/article/17/6/set-path-linux>.

Problem:

- One or more of the conda packages is/are incompatible
- `*conda create*` command fails to install packages but conda has installed correctly

Solution:

- Use the environment YAML file to install the required packages with the versions that have been tested. The YAML file can be found in the Github repository and it is named `*te_annot.yml*`. To create an environment with packages specified in a YAML file type and run the following command: `*conda env create --file te_annot.yml*`

Problem:

- Cannot download the github repository

Solution:

- Check that `git` is installed in your system by typing `which git`. Most computers come with `git` installed in their system from the manufacturer. If this is the case, a path with a location for git is shown. If it is under a miniconda/conda environment, make sure you call it from the environment in which it was installed. If you do not have git installed in your system (i.e. `which git` returns nothing), you can use `conda` to install it by running `conda install git`.

# References

[[1] R Core Team, *R: A Language and Environment for Statistical Computing*. Vienna, Austria: R Foundation for Statistical Computing, 2014. [Online]. Available: http://www.R-project.org/](https://www.zotero.org/google-docs/?oHmEo7)

[[2] A. Larsson, ‘AliView: a fast and lightweight alignment viewer and editor for large datasets’, *Bioinforma. Oxf. Engl.*, vol. 30, no. 22, pp. 3276–3278, Nov. 2014, doi: 10.1093/bioinformatics/btu531.](https://www.zotero.org/google-docs/?oHmEo7)

[[3] C. Notredame, D. G. Higgins, and J. Heringa, ‘T-Coffee: A novel method for fast and accurate multiple sequence alignment’, *J. Mol. Biol.*, vol. 302, no. 1, pp. 205–217, Sep. 2000, doi: 10.1006/jmbi.2000.4042.](https://www.zotero.org/google-docs/?oHmEo7)

[[4] ‘T-Coffee: a web server for the multiple sequence alignment of protein and RNA sequences using structural information and homology extension | Nucleic Acids Research | Oxford Academic’. https://academic.oup.com/nar/article/39/suppl_2/W13/2505784 (accessed Sep. 07, 2021).](https://www.zotero.org/google-docs/?oHmEo7)

[[5] T. Wicker *et al.*, ‘A unified classification system for eukaryotic transposable elements’, *Nat. Rev. Genet.*, vol. 8, no. 12, pp. 973–982, Dec. 2007, doi: 10.1038/nrg2165.](https://www.zotero.org/google-docs/?oHmEo7)

[[6] W. Li and A. Godzik, ‘Cd-hit: a fast program for clustering and comparing large sets of protein or nucleotide sequences’, *Bioinforma. Oxf. Engl.*, vol. 22, no. 13, pp. 1658–1659, Jul. 2006, doi: 10.1093/bioinformatics/btl158.](https://www.zotero.org/google-docs/?oHmEo7)

[[7] S. F. Altschul, W. Gish, W. Miller, E. W. Myers, and D. J. Lipman, ‘Basic local alignment search tool’, *J. Mol. Biol.*, vol. 215, no. 3, pp. 403–410, Oct. 1990, doi: 10.1016/S0022-2836(05)80360-2.](https://www.zotero.org/google-docs/?oHmEo7)

[[8] J. Mistry *et al.*, ‘Pfam: The protein families database in 2021’, *Nucleic Acids Res.*, vol. 49, no. D1, pp. D412–D419, Jan. 2021, doi: 10.1093/nar/gkaa913.](https://www.zotero.org/google-docs/?oHmEo7)

[[9] M. Blum *et al.*, ‘The InterPro protein families and domains database: 20 years on’, *Nucleic Acids Res.*, vol. 49, no. D1, pp. D344–D354, Jan. 2021, doi: 10.1093/nar/gkaa977.](https://www.zotero.org/google-docs/?oHmEo7)

[[10] S. Lu *et al.*, ‘CDD/SPARCLE: the conserved domain database in 2020’, *Nucleic Acids Res.*, vol. 48, no. D1, pp. D265–D268, Jan. 2020, doi: 10.1093/nar/gkz991.](https://www.zotero.org/google-docs/?oHmEo7)

[[11] P. Rice, I. Longden, and A. Bleasby, ‘EMBOSS: the European Molecular Biology Open Software Suite’, *Trends Genet. TIG*, vol. 16, no. 6, pp. 276–277, Jun. 2000, doi: 10.1016/s0168-9525(00)02024-2.](https://www.zotero.org/google-docs/?oHmEo7)

[[12] R. D. Finn *et al.*, ‘The Pfam protein families database’, *Nucleic Acids Res.*, vol. 38, no. Database issue, pp. D211–D222, Jan. 2010, doi: 10.1093/nar/gkp985.](https://www.zotero.org/google-docs/?oHmEo7)

[[13] A. F. Smit, R. Hubley, and P. Green, *RepeatMasker*. [Online]. Available: http://repeatmasker.org](https://www.zotero.org/google-docs/?oHmEo7)

[[14] M. A. Grohme *et al.*, ‘The genome of Schmidtea mediterranea and the evolution of core cellular mechanisms’, *Nature*, vol. 554, no. 7690, pp. 56–61, Feb. 2018, doi: 10.1038/nature25473.](https://www.zotero.org/google-docs/?oHmEo7)

[[15] N. C. for B. Information, U. S. N. L. of M. 8600 R. Pike, B. MD, and 20894 Usa, *Display BLAST search results with custom output format*. National Center for Biotechnology Information (US), 2021. Accessed: Apr. 28, 2021. [Online]. Available: https://www.ncbi.nlm.nih.gov/books/NBK569862/](https://www.zotero.org/google-docs/?oHmEo7)

[[16] K. Katoh and D. M. Standley, ‘MAFFT multiple sequence alignment software version 7: improvements in performance and usability’, *Mol. Biol. Evol.*, vol. 30, no. 4, pp. 772–780, Apr. 2013, doi: 10.1093/molbev/mst010.](https://www.zotero.org/google-docs/?oHmEo7)

[[17] R. C. Edgar, ‘MUSCLE: a multiple sequence alignment method with reduced time and space complexity’, *BMC Bioinformatics*, vol. 5, no. 1, p. 113, Aug. 2004, doi: 10.1186/1471-2105-5-113.](https://www.zotero.org/google-docs/?oHmEo7)

[[18] F. Sievers and D. G. Higgins, ‘Clustal Omega, accurate alignment of very large numbers of sequences’, *Methods Mol. Biol. Clifton NJ*, vol. 1079, pp. 105–116, 2014, doi: 10.1007/978-1-62703-646-7_6.](https://www.zotero.org/google-docs/?oHmEo7)

[[19] R. Hubley, T. J. Wheeler, and A. F. A. Smit, ‘Accuracy of multiple sequence alignment methods in the reconstruction of transposable element families’, Aug. 2021. doi: 10.1101/2021.08.17.456740.](https://www.zotero.org/google-docs/?oHmEo7)

[[20] J. Storer, R. Hubley, J. Rosen, T. J. Wheeler, and A. F. Smit, ‘The Dfam community resource of transposable element families, sequence models, and genome annotations’, *Mob. DNA*, vol. 12, no. 1, p. 2, Jan. 2021, doi: 10.1186/s13100-020-00230-y.](https://www.zotero.org/google-docs/?oHmEo7)

[[21] I. R. Arkhipova, ‘Distribution and Phylogeny of Penelope-Like Elements in Eukaryotes’, *Syst. Biol.*, vol. 55, no. 6, pp. 875–885, Dec. 2006, doi: 10.1080/10635150601077683.](https://www.zotero.org/google-docs/?oHmEo7)

[[22] E. B. Chuong, N. C. Elde, and C. Feschotte, ‘Regulatory activities of transposable elements: from conflicts to benefits’, *Nat. Rev. Genet.*, vol. 18, no. 2, Art. no. 2, Feb. 2017, doi: 10.1038/nrg.2016.139.](https://www.zotero.org/google-docs/?oHmEo7)

[[23] T. Flutre, E. Duprat, C. Feuillet, and H. Quesneville, ‘Considering Transposable Element Diversification in De Novo Annotation Approaches’, *PLOS ONE*, vol. 6, no. 1, p. e16526, Jan. 2011, doi: 10.1371/journal.pone.0016526.](https://www.zotero.org/google-docs/?oHmEo7)

[[24] R. J. Craig, I. A. Yushenova, F. Rodriguez, and I. R. Arkhipova, ‘An ancient clade of Penelope-like retroelements with permuted domains is present in the green lineage and protists, and dominates many invertebrate genomes’, *bioRxiv*, p. 2021.04.23.441226, Apr. 2021, doi: 10.1101/2021.04.23.441226.](https://www.zotero.org/google-docs/?oHmEo7)

[[25] E. A. Gladyshev and I. R. Arkhipova, ‘Telomere-associated endonuclease-deficient Penelope-like retroelements in diverse eukaryotes’, *Proc. Natl. Acad. Sci.*, vol. 104, no. 22, pp. 9352–9357, May 2007, doi: 10.1073/pnas.0702741104.](https://www.zotero.org/google-docs/?oHmEo7)

[[26] A. Cervera and M. de la Peña, ‘Small circRNAs with self-cleaving ribozymes are highly expressed in diverse metazoan transcriptomes’, *Nucleic Acids Res.*, Mar. 2020, doi: 10.1093/nar/gkaa187.](https://www.zotero.org/google-docs/?oHmEo7)

[[27] J. Cappello, K. Handelsman, and H. F. Lodish, ‘Sequence of Dictyostelium DIRS-1: an apparent retrotransposon with inverted terminal repeats and an internal circle junction sequence’, *Cell*, vol. 43, no. 1, pp. 105–115, Nov. 1985, doi: 10.1016/0092-8674(85)90016-9.](https://www.zotero.org/google-docs/?oHmEo7)

[[28] Y. C. Ribeiro *et al.*, ‘Study of VIPER and TATE in kinetoplastids and the evolution of tyrosine recombinase retrotransposons’, *Mob. DNA*, vol. 10, no. 1, p. 34, Aug. 2019, doi: 10.1186/s13100-019-0175-2.](https://www.zotero.org/google-docs/?oHmEo7)

[[29] S. Ou *et al.*, ‘Benchmarking transposable element annotation methods for creation of a streamlined, comprehensive pipeline’, *Genome Biol.*, vol. 20, no. 1, p. 275, Dec. 2019, doi: 10.1186/s13059-019-1905-y.](https://www.zotero.org/google-docs/?oHmEo7)

[[30] W. Xiong, L. He, J. Lai, H. K. Dooner, and C. Du, ‘HelitronScanner uncovers a large overlooked cache of Helitron transposons in many plant genomes’, *Proc. Natl. Acad. Sci. U. S. A.*, vol. 111, no. 28, pp. 10263–10268, Jul. 2014, doi: 10.1073/pnas.1410068111.](https://www.zotero.org/google-docs/?oHmEo7)

[[31] W. Bao and J. Jurka, ‘Homologues of bacterial TnpB_IS605 are widespread in diverse eukaryotic transposable elements’, *Mob. DNA*, vol. 4, no. 1, p. 12, Apr. 2013, doi: 10.1186/1759-8753-4-12.](https://www.zotero.org/google-docs/?oHmEo7)

[[32] J. Thomas and E. J. Pritham, ‘Helitrons, the Eukaryotic Rolling-circle Transposable Elements’, *Microbiol. Spectr.*, vol. 3, no. 4, Jul. 2015, doi: 10.1128/microbiolspec.MDNA3-0049-2014.](https://www.zotero.org/google-docs/?oHmEo7)

[[33] K. K. Kojima and J. Jurka, ‘Crypton transposons: identification of new diverse families and ancient domestication events’, *Mob. DNA*, vol. 2, no. 1, p. 12, 2011, doi: 10.1186/1759-8753-2-12.](https://www.zotero.org/google-docs/?oHmEo7)

1. if `wget` is not installed in your system, it can be installed using conda: `*conda install wget*`. It is recommended to install wget at the “base” level as this is a tool that is central to your system and not needed for a specific environment. [↑](#footnote-ref-1)
