# Supplementary material for: A beginner’s guide to manual curation of transposable elements
Source: Mob DNA. 2022 Mar 30;13:7. doi: 10.1186/s13100-021-00259-7 (PMC8969392; doi:10.1186/s13100-021-00259-7)
Supplement: Supplementary file 2 — Additional file 2. Videocast showing how to run “make_fasta_from_blast” and how to make multiple sequence alignments. [file 13100_2021_259_MOESM2_ESM.zip › Video1.docx]

# Transcript – Additional File 2 – Video 1.

00:00:06

In this video I'm going to show you how to use the script make fasta from blast and to do an alignment.

00:00:15

To retrieve sequences from the genome based on a query sequence and extend to either flank, five prime and three prime end.

00:00:27

And after that we're going to make an alignment. I've created a directory called, “fam4”.

00:00:34

In which I have placed the dummy chromosome provided to you with the github repository.

00:00:44

And I've also extracted one of the families that we are going to use to do the manual curation process.

00:00:55

The script “make fasta from blast” takes in 4 arguments.

00:01:05

The first one is the genome, which is this “chr_dummy.fasta”, we're going to use.

00:01:14

The second argument is the “fasta.in” which is the query sequence that we're going to use to start the blast.

00:01:23

The third argument is the minimum length.

00:01:25

And the 4th element is the flank. So minimum length and flank refers to distances in nucleotides. “Minimum length” is the minimum length required for heat to be included in the output fasta file.

00:01:43

And the flank is how much do we want to extend to either the three prime end and the five prime end, simultaneously. So from the hit, if hit goes between 100 and 200 and I say "flank 50”, the actual sequences that I'm going to collect is between 50 and 250.

00:02:04

Because this script uses blast, we're required to generate the blast databases. Fortunately, this script does it itself, so if it doesn't find the database files, it will generate it.

00:02:25

The same with the genome length, which is also required by the bed tools function that is embedded in the script.

00:02:34

Now to access both blast bed tools and samples which are needed in this script.

00:02:40

I will need to access the conda environment that I've created to contain all those softwares.

00:02:54

There are instructions how to create the conda environment in the supplementary materials.

00:03:02

So now I hope we are ready to start.

00:03:10

So I can say I can then type the first argument, which is the genome.

00:03:16

The second element, which is a prospective LTR element.

00:03:22

For minimal length now I have various options. I can say, what is the actual minimum I want, for example 500 basis or I can type 0.

00:03:34

As it is explained here in the help, if I type 0 then the minimum length is going to be set to half the length of the query, so this is very useful because then we are never anchored to just one value.

00:03:51

And in terms of flanks, I'm going to extend 500 bases to each side.

00:03:56

Then I'm going to click enter.

00:04:00

As expected, the blast database did not exist, so the script is running “makeblastdb” and sometimes, depending on the size of your genome, this can take a bit of time.

00:04:16

It generated the database. It also says that it cannot find the genome length, so it's making it and then at the end it's telling me that the minimum length applied to the hits in blast is 2479, that it extended each location 500 basis in each direction and that disconnected at total 14 sequences from the genome.

00:04:43

We can see now all the files that were generated and these are the database files as well as some more accessory files to generate the length of the chromosomes. And this is the output file that contains all the all the hits from the genome in a multi fasta format.

00:05:05

We can now use that multi fasta file to generate a multiple sequence alignment. We're gonna do that with the software Mafft.

00:05:16 – all of them –5 seconds

Sequence alignment and it will take as input.

00:05:21

The multi fasta that we got from running the “make fasta from blast” and we redirect the output.

00:05:31

I'm going to call it “ltr-fam4.aln.fa”. It's fairly quick to run even when you have a number of sequences. I think up to 0-60 sequences will be OK, but also depend on the length of each of those sequences.

00:05:55

This is a file with the aligned sequences that we will be able to open into an alignment viewer such as.
