# Supplementary material for: A beginner’s guide to manual curation of transposable elements
Source: Mob DNA. 2022 Mar 30;13:7. doi: 10.1186/s13100-021-00259-7 (PMC8969392; doi:10.1186/s13100-021-00259-7)
Supplement: Supplementary file 3 — Additional file 3. Videocast showing the process of manual curation of a multiple sequence alignment from a LTR family. [file 13100_2021_259_MOESM3_ESM.zip › Video2.docx]

# Transcript – Additional File 3 – Video 2.

00:00:06

Once we have generated the alignment, we can visualise it using for example AliView.

00:00:15

I've already have AliView open so I am going to open the alignment file that was generated previously with Mafft.

00:00:24

I go to file, open file and this opens a window to navigate through my computer system.

00:00:32

And one quick way of accessing the files is by clicking “date modified”, so the latest one modified will be at the top.

00:00:43

This is the one I've generated with Mafft in the previous step.

00:00:45

So I'm going to open it.

00:00:48

The alignment will open automatically at the beginning on the 5’ end.

00:00:54

As the sequences are presented and normally, we will see that there's not much conservation at the beginning because this is the flanks that we extended on the hits, and because we were trying to.

00:01:07

Capture as much transposable element sequence as possible in any given genomic location.

00:01:14

Inevitably, and we want to do this part of these sequences are not going to be part of the transposable element, because what we are going to want we're going to want to find the boundary of where that transposable element starts.

00:01:28

So we can use the navigation bar here at the bottom to scroll.

00:01:33

And downstream if you want and we will see that at some point the block of conservation appears

And here you can see that here on the left hand side there is very little conservation and here on the right hand side the conservation starts, so

00:01:49

I think we can safely say that perhaps somewhere.

00:01:53

Here is where the sequence our team is going to start on this team, so LTR elements have a canonical start on their LTR subparts that is.

00:02:04

TGT could be TT GT, but it's always a combination of those two nucleotides and because the LTR subparts are repeats at the start and at the end of the element we will see the reverse complementary of this one at the other end, which we're going to visualise in in a little while.

00:02:27

So we can select this column and then using shift, alt and the left arrow we can then select everything that is to the left of that column and we can either right click and delete select sequences or you can.

00:02:47

Use the shortcut, which is a command backspace to delete all those sequences. So I've deleted those the all the columns to the left of my selected column, and now I can see that at the very beginning of my sequence alignment.

00:03:05

And the first row is a very highly. The first column is a highly conserved T.

00:03:11

We also have some insertions here of some sequences that perhaps are not very well conserved and we're going to delete those ones later on.

00:03:20

The next step is to go to the other end of the alignment. You see all those insertions, but let's not worry about those ones at the moment. We will take care of them later on.

00:03:31

As I progress, we see very very lot of conservation there. This is quite a nice element, quite a nice family, but at some point that conservation block is going to end and I'm going to find very divergent sequences and what happens is that will indicate the end of the transposable element conservation.

00:03:53

And that is happening at about here.

00:03:55

So I didn’t extend very much at the beginning using “make_fasta_from_blast”, I only used about 1000 bases and that was enough because for this particular element.

00:04:06

You can see that if I scroll even further downstream there is lots of lack of conservation and this is how it looks but then, as what happens with the 5’ end,

00:04:19

All of a sudden we scroll up and down and we can see that there is a very well conserved block here.

00:04:23

And as I predicted from the beginning of the element that we had TGT, then we have the reverse complement here, which is ACA.

00:04:32

So I'm quite confident that perhaps the TE maybe ends there could potentially end there. I am just going to include this particular row as the last row of the element.

00:04:44

In similar ways we did for the five prime end, I can select this column and using shift, alt and the right arrow I can select all the columns to the right of that particular column and I can delete those sequences.

00:05:01

Now we have the boundaries of our transposable element family defined.

00:05:07

So we can proceed to delete large insertions that will not contribute to our consensus sequence.

00:05:18

The first thing to notice is that we can delete sequences by clicking, for example on a row. So I could potentially delete all these two sequences that I don't feel are true representatives of the of the family.

00:05:31

This one has a very large gap and the other one has a lot of nucleotides that do not seem to align.

00:05:37

So we could delete these ones by clinking them both and then doing the same them by deleting the selected sequences.

00:05:47

And now we will see that some parts will show gaps that do not have any sequences, so empty sequences, that were previously, ehem the spaces that were previously occupied by those sequences such as this example.

00:06:03

So we can manually delete that gap. Or the other thing we can do is we can rerun the alignment and AliView comes with its own aligner, which also uses Mafft and we can specify that in the preferences so I can just click align.

00:06:16

And tell AliView to realign everything and that is running the alignment.

00:06:24

And it will automatically display it and we can see that we don't have that gap anymore. But we do have some other little gaps there. I could see here there's.

00:06:35

A little one gap. So I would like to delete that one. We can do this in the same way I'm going to use the shortcut now, which is CMD backspace to delete that column.

00:06:45

I'm going scrolling like that through the alignment is the way that we remove those unwanted sequences. It's not any sequence that we remove, but those things that are not well conserved.

00:06:56

So for example this insertion here. I don't believe this will conserve is not going to contribute to the consensus, so I get rid of it.

00:07:05

And the objective of this exercise is to remove all the sequences that perhaps are not going to contribute to the consensus.

00:07:15

There are not that many. Here you can see that this is very very well conserved, perhaps a couple of nucleotides here and there. But it's a very easy example.

00:07:25

To get us started with.

00:07:30

Here there's another.

00:07:33

Single insertion.

00:07:38

Scroll, scroll scroll.

00:07:41

Here we have quite a large insertion, which I'm also going to remove.

00:07:48

Here is there's a lot less conservation here, so we have a lot of …

00:07:53

A bit of a large insertion here. I'm going to zoom out a little here so to have a better view of that area

00:08:00

And we lose the resolution. I'm going to delete that column and in here I am going to make the decision of removing all the sequences that produce this insertion here.

00:08:21

That is the bit that I'm going to remove from. I am also going to remove these ones and this other.

00:08:25

I could potentially remove this last sequence, this last row. The sequence that is presented in the last row.

00:08:35

They are probably not a very good, very good representative here of the family.

00:08:41

But for the purpose of this this example we're going to keep it. This is a very large insertion, so we'll have to zoom out quite a lot to be able to delete it.

00:08:55

As you can see this is a very manual process. And quite labour intensive, but we do get a very good understanding of what the sequence looks like.

00:09:08

I think I will delete this one. At the end, because there's just so many places where that is not contribution to the consensus.

00:09:18

I will need now to apply a realignment step, we’ll still have some individuals insertions.

00:09:29

And I think we are nearly to the end because I have realigned a couple of times, I will have to have a look through the alignment.

00:09:41

To make sure that there is no big gaps.

00:09:47

We can keep scrolling. We can see that it is quite a robust consensus.

00:09:53

Quite a robust alignment.

00:10:00

And I would say that now our family is finally curated.

00:10:04

So we have lost three sequences, three input sequences to this alignment. But we have a set of sequences that will contribute strongly to the consensus.

00:10:14

Before we leave the most important part of this work is to save our work. So we are going to go to “File” , then “Save as FASTA”, and I am going to change the name of this file to call it “edit”

00:10:28

So I am not re-writing to the same file in case I want to access the original MAFFT alignment. I am going to save is as “my_edit”
