# Supplementary material for: A beginner’s guide to manual curation of transposable elements
Source: Mob DNA. 2022 Mar 30;13:7. doi: 10.1186/s13100-021-00259-7 (PMC8969392; doi:10.1186/s13100-021-00259-7)
Supplement: Supplementary file 4 — Additional file 4. Videocast showing the process of manual curation of a multiple sequence alignment from a LINE family. [file 13100_2021_259_MOESM4_ESM.zip › Video3.docx]

# Transcript – Additional File 4 – Video 3.

00:00:04

Now go to AliView, then “Open file”.

00:00:10

I need to change directories because I've created a new directory for this work.

00:00:16

And I have here the last file that was created, which was my alignment file.

00:00:23

And this is a LINE element, so we are expecting, I know this is a LINE element already.

00:00:30

The point of this video is to show you how LINE elements look in a sequence alignment file.

00:00:40

As with the LTR family, that we did previously, his one is also not very well conserved at this end. In general, LINES and and Penelope elements are not going to be very well conserved on the five prime end.

00:01:00

So it might not be that easy to find that block of conservation that we saw before …

00:01:04

For the LTR element, however, there will be a block of conservation from the three prime end. So for this particular purpose, I would suggest we start from the three prime end.

00:01:16

You can see my bar here at the very end, and I'm going to scroll towards the five prime end until I can find a very conserved block of sequences.

00:01:27

And that will indicate the end of my transposable element family in this point.

00:01:36

So I would say that perhaps this is the first column that is not very well conserved.

00:01:41

Uh, I can select that column and with the shift alt and the right arrow in my keyboard I can select all the columns to the right and I can delete them.

00:01:53

And we can now start to get rid of the insertions. We are not going to worry too much about the five prime end for now.

00:02:03

We're going to delete insertions that are not going to contribute towards our consensus sequence.

00:02:13

And again, this is a sort of like a labour intensive job, but it's necessary and it will give us a better insight of how our element looks like.

00:02:30

There's a very large insertion there, so I'm going to zoom out.

00:02:34

Recenter my element.

00:02:37

Select all the columns and then delete them.

00:02:41

There we go.

00:02:43

We can keep scrolling either with the bar or step by step.

00:02:48

It's another insertion here.

00:02:52

And so on and so forth. Now you would start seeing, I hope you can appreciate, that as I move towards the left hand side and towards the five prime end, the sequences at the bottom of the alignment become less and less conserved.

00:03:11

So I'm now moving to the right, you can see. There's quite a good block of conservation here and as I move to the left these sequences start to be more poorly conserved.

00:03:23

This is very characteristic of LINE and Penelope elements. As we move towards the five prime end, the conservation drops.

00:03:32

And it will not drop for all of the copies at the same time. It will drop for some because some copies will be more complete than others, so the copies that are more complete in this case.

00:03:44

Are the ones that are the very top.

00:03:46

And the ones that are incomplete are the ones at the very bottom. They are either incomplete or the how they have decayed.

00:03:56

There are different ways of resolving this problem, so we perhaps don't want all of these sequences to contribute to the alignment.

00:04:06

So what we can do is we can decide a boundary where we want to stop considering those sequences, so we can.

00:04:16

We're going to visualise it at the very end, and then we're going to decide where we're going to draw that boundary because there's a lot of insertions here.

00:04:26

Uhm no, we still need to.

00:04:29

Get rid of. Although very likely that we're going to get rid of these insertions once we remove the sequences from the bottom.

00:04:38

But we also have to at some point stop this sequence, and we're not going to keep all the bases that are towards the five prime end. We're going to define a boundary at some point.

00:04:51

But I'm still pretty happy with this block of conservation here of these sequences.

00:04:56

So I'm going to continue towards the left.

00:04:59

Towards the left towards the left you could spend time deleting these ones, but it's likely that we're going to end up.

00:05:06

Removing them in a more automatic way later on, but for the sake of example.

00:05:12

We're going to remove them.

00:05:15

Like this

00:05:18

And I guess it's clearer here. If you look at the top half of the sequences compared to the top bottom that the conservation is very very very different.

00:05:26

The ones at the top are very well conserved and the ones at the bottom are not.

00:05:33

And as we progress further and further towards the five prime end, then these ones become even less conserved. So there are more and more sequence that are less conserved.

00:05:43

After a particular point where that conservation is going to drop, so I would be perhaps quite happy to stop it even before that.

00:05:51

Perhaps around …

00:05:54

For this, for this example, we're going to stop them around here, and we're going to get rid of everything else that is upstream of that.

00:06:05

Now we can see that there's still a lot of the bottom sequences are however, divergent. So what I'm going to do is I am going to.

00:06:15

Remove all of these sequences from the alignment so I could.

00:06:21

I could delete only from here to the left for example and get rid of only those sequences. Or we could delete them all.

00:06:31

So this would only delete this block, perhaps we should do that.

00:06:39

Let's see how we can do that. Select these 4 columns and then shift, Alt on the left arrow will select everything that is on the left. Now instead of deleting the residues now what we are going to do is we're going to.

00:06:57

Clear them.

00:06:58

We're going to make little hyphens there if it were an alignment, because if we delete them then all the sequences that are here on the right.

00:07:06

We're going to shift to the left and we don't want that. We want to keep these sequences where they are on the right.

00:07:13

So how do we do this?

00:07:18

We go to edit.

00:07:22

And we select “clear selected bases” not “delete”, we select “clear selected basis”.

00:07:29

And that will remove those sequences without altering the order of these ones on the right hand side.

00:07:40

There are some gaps here.

00:07:43

That I'm going to remove by hand.

00:07:52

And I will be perhaps pretty happy that this is going to make a good consensus.

00:07:58

And perhaps one more thing we could do is that because as we progress towards the five prime end, there's more sequences that appear more degraded. I could probably do the same exercise that I did before.

00:08:11

And at this level, perhaps around here.

00:08:25

That's probably OK. Yeah, that is probably OK, so I'm going to stick with this with this alignment.

00:08:32

You could potentially try to realign as well as we did before, aligned and realign everything again, but I suspect that these blocks here might change position so we don't really know what would happen. We could probably try it out. I'm going to first save this.

00:08:49

Uhm, this file.

00:08:55

There we go.

00:08:57

And that's it.

00:09:00

One more thing we can do now is we can go back to our Unix screen.

00:09:06

We can work with our edited file and we can generate a consensus from there.

00:09:12

“Cons” is the tool that comes with EMBOSS and it generates a consensus.

00:09:19

Here is the help.

00:09:20

We can give it the input sequence under this flag and the output sequence name under this flag, and it has a number of different functions different options that we can select in terms of plurality and identity, and I recommend you read the manual carefully so you might want to change those things as you as you work.

00:09:43

I'm going to list the files so I don't make any mistakes.

00:09:57

So what the making a consensus means is that it's going to make one sequence based on all the multi sequences that we had in the alignment. That is a summary of all the sequences appearing there.

00:10:15

So now we can see how our consensus looks like.

00:10:20

And you will see sometimes that the consensus might have some “Ns”, like it happens here, because if there is a lot of variation on a particular column of the alignment, it won't be able to call the base with any certainty.

00:10:34

So it will put “Ns” and sometimes you have to have lots of Ns, so this is at the very beginning. Lots and lots of Ns because it doesn't.

00:10:41

It doesn't know because there's so much variability at this end that it doesn't know what to put.

00:10:47

We could potentially trim this or we can change the plurality.

00:10:49

when we call “cons” and it will make decisions based on those on those flags.

00:11:01

And this represents the end of the manual creation of our LINE family.
