# Supplementary material for: A beginner’s guide to manual curation of transposable elements
Source: Mob DNA. 2022 Mar 30;13:7. doi: 10.1186/s13100-021-00259-7 (PMC8969392; doi:10.1186/s13100-021-00259-7)
Supplement: Supplementary file 5 — Additional file 5. Videocast showing how to split a multiple sequence alignment in AliView. [file 13100_2021_259_MOESM5_ESM.zip › Video4.docx]

# Transcript – Additional File 5 – Video 4.

00:00:07

In this video we are going to show you how to split an alignment.

00:00:12

That had picked up two different groups or two different subfamilies as a result of a blast search.

00:00:21

I have open AliView

00:00:24

Which is my preferred Multiple sequence alignment viewer.

00:00:31

And the alignment that I'm showing you is a result of having run a blast with a query sequence that is a prospective TE family against the genome, so this could have been for example the result of running “make_fasta_from_blast” that we explained in the …

00:00:51

supplementary material and in the main part of the text of the document.

00:01:00

I'm going to scroll towards the right so you can see the full length of the alignment because we have extended.

00:01:08

towards the flanks.

00:01:11

We can see that towards the five frame end and we will see the same towards the three prime end,

00:01:17

That there is quite a low level of conservation in these areas. We can see this because.

00:01:23

We don't see any alignment.

00:01:26

But at some point we start seeing that half of these sequences.

00:01:33

Show a certain level of conservation. They show that they are very well aligned, as we can see in this particular block, in the top part of the window.

00:01:44

However, in the bottom part of the window, so these sequences, show very little conservation, very little conservation with respect to the top sequences.

00:01:56

But nonetheless they have a level of similarity.

00:02:04

With the rest.

00:02:05

Of the sequences and that is why.

00:02:07

The blast hits were retained in this particular example.

00:02:14

So because of they were retianed because they are also.

00:02:18

Aligning sort of in blocks.

00:02:20

Can't start to think about the prospect of these two being independent, two very different groups of sequences that could result in two families.

00:02:33

So in order to curate these ones, I am going to attempt to split this alignment and create two transposable element families.

00:02:43

From each of these two distinct groups.

00:02:48

As a third step, I am going to trim the ends of the alignment.

00:02:55

So we have shown before how this is done.

00:02:59

I'm going to do this very quickly by selecting and removing the columns.

00:03:04

I am going to quickly travel to the three prime end where you will always also going to be able to see lack of conservation and where the end of my …

00:03:18

Conserved alignment is.

00:03:21

So I'm going to remove this section as well. That is the first part that we do.

00:03:27

And now I am going to select the sequences that I want to transfer to a different file.

00:03:35

And those are going to be the ones that do not align well with the top block of the sequences.

00:03:45

So by selecting the sequences from the left hand navigation side navigation bar, I can then right click and copy them as fasta.

00:03:58

I can then select to create.

00:04:01

Uhm, to create a new file.

00:04:05

Which opens a window completely blank.

00:04:12

And if I just paste the sequences here.

00:04:16

The same ones that come that's going to transfer the selected sequences from this from the top window into my new window.

00:04:27

The first thing I want to do is perhaps save this file.

00:04:32

So I can save it and give it a name.

00:04:46

The next thing I want to do is … I want to re align it because before they were aligned with respect of the whole.

00:04:53

All of the whole of the sequences, and that's why they end up split into blocks.

00:04:57

So for this second alignment I can try to align them again, so I'm going to use a “realign everything” tool that comes in with AliView, and in this case it's running mafft. .

00:05:09

Now we can see that.

00:05:13

The alignment is slightly better.

00:05:18

But it is again. There's quite a lot of sequence here that is some that is lacking conservation and we need to then trim the edges again, which I'm going to start doing right now.

00:05:40

The three prime end of this particular elements seem to have been quite well trimmed.

00:05:48

So now we can see that we have two different, uh, the bottom one becomes a different family than the front one.

00:05:57

In order to finalise the curation of the first family.

00:06:05

I can then collect the top sequences and repeat the procedure. I'm going to copy them.

00:06:16

I'm going to open a new file.

00:06:19

I'm going to paste them.

00:06:22

And I'm going to align them.

00:06:26

So now we have two very different sequences.

00:06:30

That come from the original, from the same original multiple sequence alignment.

00:06:35

Notice that the larger family has 7500 … .

00:06:41

Positions in this particular MSA, whereas the shorter version has only 1322 positions.

00:06:52

I would suspect that the shorter version could be the non autonomous version of the larger one.

00:06:59

And it would be lacking some of the key protein domains that will allow the autonomous version of the transposable element family. To be able to jump independently.

00:07:15

When you finish creating any of these alignments, do not forget to save them into a file, and later on you can generate a consensus from each of them.

00:07:26

Thank you for watching this video and I hope you have enjoyed it.
