# Supplementary material for: A beginner’s guide to manual curation of transposable elements
Source: Mob DNA. 2022 Mar 30;13:7. doi: 10.1186/s13100-021-00259-7 (PMC8969392; doi:10.1186/s13100-021-00259-7)
Supplement: Supplementary file 6 — Additional file 6. Videocast showing a selection of Unix commands to assist the handling of FASTA flies. [file 13100_2021_259_MOESM6_ESM.zip › Supplementary_Video_1.docx]

# Transcript – Additional File 6 – Supplementary Video 1.

00:00:05

I have placed in this file in this folder.

00:00:11

The other reference genome, which is chromosome dummy Fasta.

00:00:16

The city hit output, so this is after reducing redundancy from the RepeatModeller output and a text file that has the names of the families that I am going to manually curate. In the previous video we've already done.

00:00:31

Uhm family 4 so we're going to do rnd-1 family 178 as mentioned earlier.

00:00:37

So one thing that we didn't address in the previous video is how do we extract a single FASTA file from

00:00:45

A multi fasta file, a single fasta sequence from a multi fasta file based on the header just all just on the header of the sequence.

00:00:55

So there are two ways of doing this. The 1st way is using grep. Grep is a tool that is built-in in all of the Unix systems.

00:01:05

And it's a word matching programme. So for example.

00:01:12

if I wanted to find the road that has rnd in the file “to_curate.txt”, I can just.

00:01:21

Say “grep” rnd from this file and it will give me that line so we can use the same principle to and we use this as a query and we can look for the sequence in this file.

00:01:35

Now what's going to happen with that?

00:01:39

Is that …

00:01:41

We'll only get the header and we want not just the header, but we want the header and sequence.

00:01:47

There is another tool from grep another flag that is called “-A 1” so “-A 1” tells Grep to give us back the line that has the match and the line that comes after.

00:02:03

But of course, our fasta, our multi Fasta file is broken every hundred or so nucleotides.

00:02:13

So we have to first convert this into a “one line” fasta. There is a little script that does this and comes with the GitHub repository and it's called …

00:02:28

“Fasta_multi_to_one” so we can convert all of the cdhit.fa into another file.

00:02:39

And now the difference between this is that this new file, instead of having the nucleotide sequences broken, it has them all in one line. So this is one line, the next one. Or this is one line. So now if we.

00:02:57

Again, use the grep -A 1 function to search.

00:03:07

The file that I've just made then will have all the full of the sequence so we can redirect this.

00:03:14

Into another file.

00:03:19

Another way is to use the tool “fasplit”. fasplit is very flexible and one of the functions that were going to use is there to split it by name. So every time it finds a new Fasta header, it will create a new file.

00:03:39

The file that we're going to split is.

00:03:43

This one. It could be the other one as well, with the original. And we're going to put that… . Actually! I'm going to make a directory called “ind_seqs” that stands for “individual sequences”, and we're going to put the output of that fasplit into that folder.

00:04:01

Now we will see that this is going to give us an error and this is because there is this character in the name of the fasta. So if I “grep” this from the file ….

00:04:21

You will see that all of them have a bar. When you are writing the name of a file, this indicates that they are directory. So we first need to ….

00:04:31

Extract that file or convert it into something else. We can do this with SED.

00:04:41

So I'm now converting it into just a single space.

00:04:57

There we go, so we can now look.

00:05:00

into this file. We can now use this file as the one that we want to split instead of the cdhit so we can repeat fasplit.

00:05:14

But with no_bar.

00:05:17

And we will put the output in “ind_seqs” and that is done. So we can now list the contents.

00:05:26

Of the “ind_seqs”….

00:05:30

Folder we will have that we have one file for each of the families that were in that “cdhit” file.

00:05:39

So these are the two ways that we can create individual sequences.

00:05:44

We are going to work only with this one for now.

00:05:49

And as we did for family four, I am going to use this.

00:05:57

To “make_fasta_from_blast”.

00:06:00

This is the genome that I'm going to use.

00:06:03

This is the fasta that I'm going to use again. I'm going to ask for at least half the size and I'm going to extend 1000 bases either side.

00:06:14

Is making the …

00:06:16

“Blastdb” auxiliary files and as well as the genome length we can see them here. The new files have been generated as well as the multi fasta here.

00:06:30

So as we did for family 4, for the next step is to align these sequences using mafft.

00:06:43

That's going to take a few seconds.

00:06:46

And after that we are going to open that file in Aliview to edit the alignment to manually curate the alignment.

00:06:57

That's done.

00:07:00

This is the mafft output.
